# DNA topoisomerase II inhibition potentiates osimertinib’s therapeutic efficacy in EGFR-mutant non–small cell lung cancer models

**DOI:** 10.1172/JCI172716

**Published:** 2024-03-07

**Authors:** Zhen Chen, Karin A. Vallega, Dongsheng Wang, Zihan Quan, Songqing Fan, Qiming Wang, Ticiana Leal, Suresh S. Ramalingam, Shi-Yong Sun

**Affiliations:** 1 Department of Hematology and Medical Oncology, Emory University School of Medicine and Winship Cancer Institute, Atlanta, Georgia, USA.; 2 Department of Pathology, The Second Xiangya Hospital, Central South University, Changsha, Hunan, China.; 3 Department of Internal Medicine, The Affiliated Cancer Hospital of Zhengzhou University, Henan Cancer Hospital, Zhengzhou, China.

**Keywords:** Oncology, Therapeutics, Apoptosis, Lung cancer, Telomeres

## Abstract

Development of effective strategies to manage the inevitable acquired resistance to osimertinib, a third-generation EGFR inhibitor for the treatment of EGFR-mutant (EGFRm) non–small cell lung cancer (NSCLC), is urgently needed. This study reports that DNA topoisomerase II (Topo II) inhibitors, doxorubicin and etoposide, synergistically decreased cell survival, with enhanced induction of DNA damage and apoptosis in osimertinib-resistant cells; suppressed the growth of osimertinib-resistant tumors; and delayed the emergence of osimertinib-acquired resistance. Mechanistically, osimertinib decreased Topo IIα levels in EGFRm NSCLC cells by facilitating FBXW7-mediated proteasomal degradation, resulting in induction of DNA damage; these effects were lost in osimertinib-resistant cell lines that possess elevated levels of Topo IIα. Increased Topo IIα levels were also detected in the majority of tissue samples from patients with NSCLC after relapse from EGFR tyrosine kinase inhibitor treatment. Enforced expression of an ectopic *TOP2A* gene in sensitive EGFRm NSCLC cells conferred resistance to osimertinib, whereas knockdown of *TOP2A* in osimertinib-resistant cell lines restored their susceptibility to osimertinib-induced DNA damage and apoptosis. Together, these results reveal an essential role of Topo IIα inhibition in mediating the therapeutic efficacy of osimertinib against EGFRm NSCLC, providing scientific rationale for targeting Topo II to manage acquired resistance to osimertinib.

## Abstract 2

DNA topoisomerase II inhibition mediates the therapeutic efficacy of osimertinib in models of EGFR-mutant non&ndash;small cell lung cancer.

## Graphical abstract

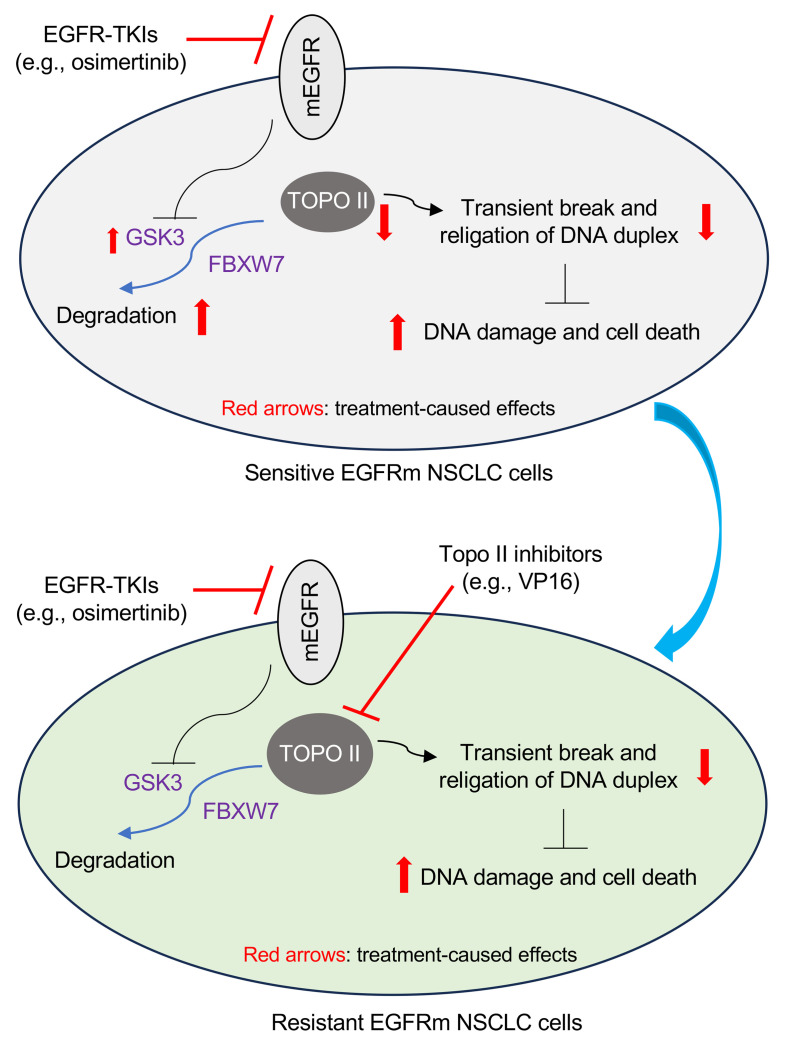

## Introduction

Lung cancer has remained the leading cause of cancer death among both men and women, and it accounts for around one-fourth of all cancer deaths worldwide despite great efforts to improve outcomes over past decades. Advances in the development of various targeted therapies and immunotherapy have substantially improved the 5-year survival rate of lung cancer in the United States, although it is still low at around 20% (1). The discovery of EGFR-activating mutations as a predictor of patient response to EGFR tyrosine kinase inhibitors (EGFR-TKIs) represented a milestone and paradigm shift in the treatment of non–small cell lung cancer (NSCLC). As the first successful targeted therapy against lung cancer, EGFR-targeted therapy has contributed to improving the quality of life and survival of patients with lung cancer.

However, the key challenge in clinical practice is the inevitable development of acquired resistance, which limits the long-term benefit of this targeted therapy. Advances in the field to battle against acquired resistance have led to the rapid development of new generations of EGFR-TKIs, from the initial first-generation (e.g., erlotinib and gefitinib) to second-generation (e.g., afatinib) and the current third-generation (e.g., osimertinib or AZD9291) agents. Third-generation EGFR-TKIs are described as mutation-selective EGFR-TKIs because of their selective and irreversible effects against EGFR-activating and T790M-resistant mutations. Among them, osimertinib is the first FDA-approved EGFR-TKI for patients with EGFR-mutant (EGFRm) NSCLC whose disease has become resistant to first-generation EGFR-TKIs through the T790M mutation as a second-line treatment and for EGFRm advanced NSCLC as a first-line treatment. Osimertinib has demonstrated impressive activity in prolonging overall survival (OS) of patients for over 3 years (2). Despite this, acquired resistance inevitably occurs to osimertinib and other third-generation EGFR-TKIs (3–5). Thus, the development of effective strategies to overcome acquired resistance to third-generation EGFR-TKIs is an urgent and critical area of unmet need in the clinic.

Conventional cytotoxic chemotherapy remains the mainstay of treatment for many types of cancer, including NSCLC. Platinum-based doublet chemotherapy has been the backbone of NSCLC treatment for decades and still represents a key therapeutic option, even in the era of modern immunotherapy (6, 7). It is also an option for patients with EGFRm NSCLC who have disease relapse from osimertinib and have no other treatment options. However, it is unclear whether there is a role for chemotherapy in managing acquired resistance to osimertinib and, if so, what the scientific rationale is for choosing the optimal chemotherapeutic agents, even though chemotherapy is used in the treatment of osimertinib-relapsed patients in real world clinical practice. In our effort toward understanding and overcoming acquired resistance to osimertinib, we found that DNA topoisomerase II (Topo II) inhibitors, doxorubicin (DXR; adriamycin) and etoposide (VP-16), but not other chemotherapeutic agents, such as cisplatin, carboplatin, and paclitaxel, synergized with osimertinib to decrease the survival of several osimertinib-resistant cell lines, accompanied by enhanced induction of apoptosis, suggesting their unique potential in overcoming acquired resistance to osimertinib.

Topo II is a well-known cancer target. Both DXR and VP-16, although used less frequently in NSCLC, act by trapping Topo II on DNA to increase the levels of Topo II–DNA covalent complexes, thereby preventing DNA replication and transcription and causing DNA single- and double-strand breaks (DSBs; or DNA damage), which in turn result in apoptosis when not adequately repaired (6, 8). It has been shown that inhibition of EZH2, a methyltransferase, sensitizes EGFRm lung tumors to Topo II inhibitors such as VP-16, suggesting that dual inhibition of EZH2 plus Topo II represents a treatment option for EGFRm tumors (9).

Therefore, we focused on demonstrating the efficacy of Topo II inhibitors, particularly VP-16, in overcoming acquired osimertinib resistance when combined with osimertinib and on defining the underlying scientific rationale. As a result, we confirmed the effects of osimertinib in combination with a Topo II inhibitor on the growth of osimertinib-resistant xenograft tumor in vivo and on delaying the emergence of acquired resistance to osimertinib. Consistently, osimertinib as well as other EGFR-TKIs decreased the levels of Topo IIα in EGFRm NSCLC cells, and this was accompanied by increased DNA damage; these effects were lost in osimertinib-resistant cell lines that possessed elevated basal levels of Topo IIα. Importantly, knockdown of *TOP2A* in osimertinib-resistant EGFRm NSCLC cell lines sensitized their responses to osimertinib, including induction of DNA damage and apoptosis, whereas enforced overexpression of *TOP2A* in sensitive EGFRm NSCLC cells conferred resistance to osimertinib. These findings demonstrate a potentially novel connection between Topo II modulation and therapeutic efficacy of EGFR-targeted therapy, thus providing a scientific rationale for targeting Topo II to delay and overcome acquired resistance to osimertinib as well as other EGFR-TKIs.

## Results

### Chemotherapeutic agents with Topo II–inhibitory activity when combined with osimertinib exhibit potentiated effects, including decreasing the survival and inducing Bim-dependent apoptosis of osimertinib-resistant cell lines.

**Figure 1:**
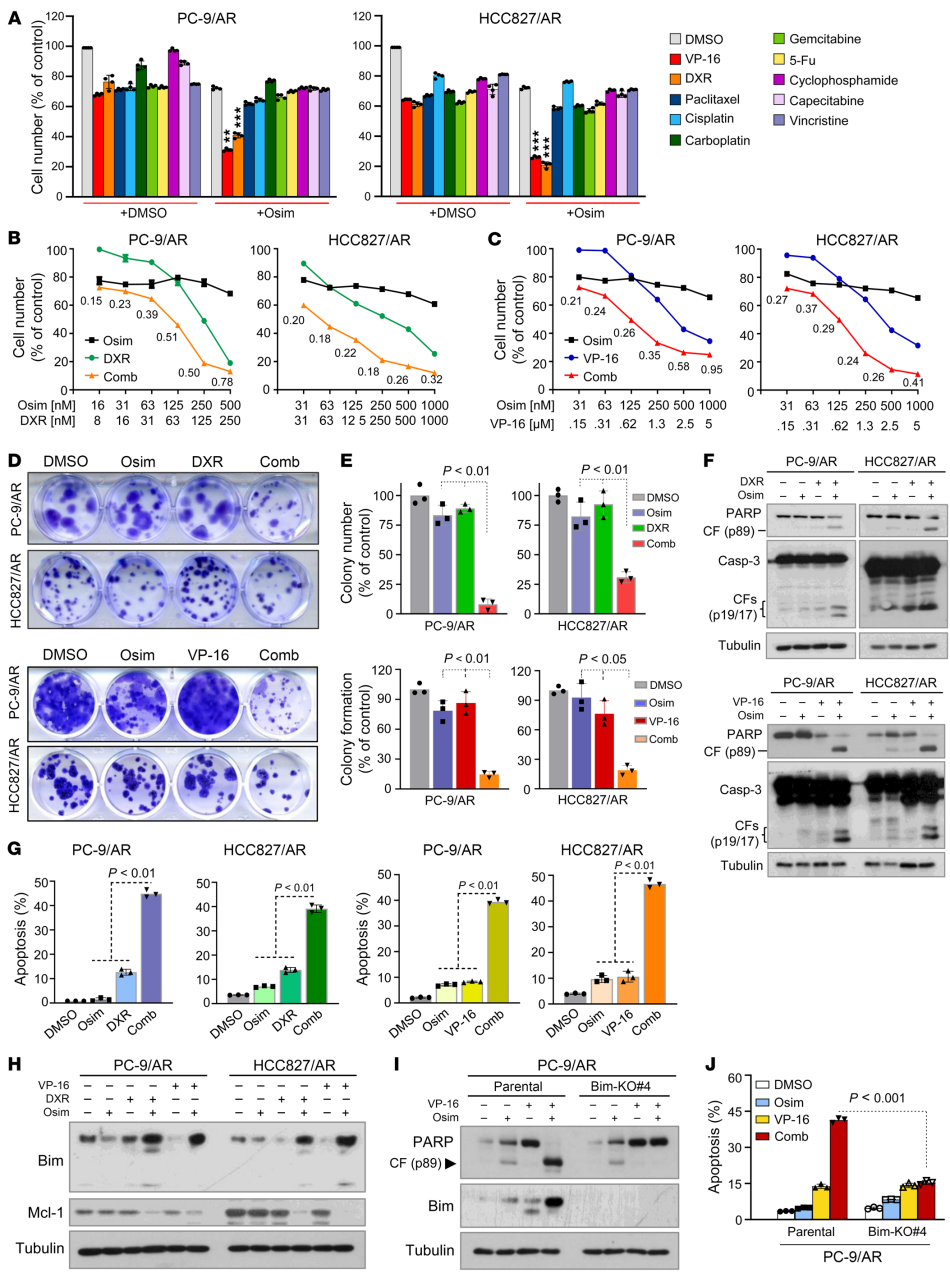
Topo II inhibitors in combination with osimertinib synergistically decrease cell survival, inhibit colony formation and growth, and induce Bim-dependent apoptosis in osimertinib-resistant EGFRm NSCLC cell lines. (**A**–**C**) The given cell lines were treated with 250 nM osimertinib (Osim), 1.25 μM VP-16, 125 nM DXR, 5 nM paclitaxel, 10 μM cisplatin, 25 μM carboplatin, 25 nM gemcitabine, 20 nM 5-FU, 25 μM cyclophosphamide, 25 μM capecitabine, or 10 nM vincristine alone or in combination (**A**) or with varied concentrations of the tested agents either alone or in combination (**B** and **C**) for 3 days. Cell numbers were then measured using the SRB assay. Data represent mean ± SD of 4 replicate determinations. **P < 0.01; ***P < 0.001 compared with each agent alone. The fixed suboptimal concentrations of the tested agents used in **A** were chosen based on their concentration-dependent survival curves. (**D** and **E**) The tested cell lines seeded in 12-well plates were treated with 50 nM osimertinib, 10 nM (PC-9/AR) or 50 nM (HCC827/AR) DXR, 150 nM VP-16, or the indicated combinations, which were repeated with fresh medium every 3 days. After 10 days, the cells were fixed, stained with crystal violet dye, imaged (**D**) and counted (**E**). Columns are mean ± SD of triplicate determinations. (**F**–**J**) The tested cell lines were exposed to 200 nM osimertinib, 100 nM (PC-9/AR) or 250 nM (HCC827/AR) DXR, 1 μM VP-16, or the indicated combinations for 48 hours (**F**, **G**, **I**, and **J**) or 16 hours (**H**). The proteins of interest were detected with Western blotting (**F**, **H**, and **I**), and apoptotic cells were detected with annexin V staining/flow cytometry (**E** and **J**). Each column represents mean ± SD of triplicate treatments. Statistical differences were conducted with 2-sided unpaired Student’s *t* test for 2 groups (**J**) or 1-way ANOVA test (**A**, **E**, and **G**) for multiple groups.

To determine whether any chemotherapeutic agents, when combined with osimertinib, enhance the decreased survival of osimertinib-resistant cells, we screened the efficacies of 10 commonly used chemotherapeutic agents combined with osimertinib, respectively, against the growth of the 2 EGFRm NSCLC cell lines with acquired resistance to osimertinib, PC-9/AR and HCC827/AR. Among them, DXR and VP-16 stood out as the only 2 agents that were significantly more effective than any agent alone, when combined with osimertinib, in decreasing the survival of these 2 cell lines (Figure 1A and Supplemental Figure 1; supplemental material available online with this article; https://doi.org/10.1172/JCI172716DS1). The combination of osimertinib with either DXR or VP-16 was synergistic in decreasing the survival of both PC-9/AR and HCC827/AR cell lines, given the combination indexes (CIs) were less than 1 (Figure 1, B and C). The colony formation assay, which allows us to repeat the treatments for a relatively long period of time, further demonstrated that the combination of osimertinib with either DXR or VP-16 was significantly more active than either single agent in inhibiting the formation and growth of PC-9/AR and HCC827/AR colonies (Figure 1, D and E). The combinations were also significantly more effective than either agent alone in the induction of apoptosis, as shown by enhanced cleavage of both PARP and caspase-3 (Figure 1F) and annexin V–positive cells (Figure 1G) in both PC-9/AR and HCC827/AR cells. In agreement with this, we also detected enhanced effects of osimertinib with DXR on decreasing the survival, inhibiting colony formation and growth, and inducing apoptosis of PC-9/GR/AR cells (Supplemental Figure 2).

Osimertinib is known to induce apoptosis via modulating Bim and Mcl-1 levels in EGFRm NSCLC cells (10). To demonstrate whether the combination of osimertinib with DXR or VP-16 results in enhanced apoptosis via altering the levels of Bim and Mcl-1, we assessed the effects of their combination on the levels of these proteins in PC-9/AR and HCC827/AR cells lines. Whereas osimertinib, DXR, or VP-16 alone did not increase Bim levels or decrease Mcl-1 levels, the combination of osimertinib with DXR or VP-16 effectively elevated Bim levels while decreasing Mcl-1 levels in these 2 cell lines (Figure 1H). Accordingly, the combination of osimertinib with VP-16 led to greater induction of PARP cleavage and an increase in annexin V–positive cells in PC-9/AR cells but not in PC-9/AR-Bim–KO cells (Figure 1, I and J). Similar results were generated in HCC827/AR-Bim KO cells (Supplemental Figure 3). These data demonstrate that the combination of osimertinib with DXR or VP-16 enhances Bim-dependent apoptosis in osimertinib-resistant cells and the inclusion of DXR or VP-16 restores the ability of osimertinib to induce Bim-dependent apoptosis in osimertinib-resistant cells.

### The combination of osimertinib with a Topo II inhibitor effectively inhibits the growth of osimertinib-resistant tumors in vivo and is well tolerated in mice.

**Figure 2:**
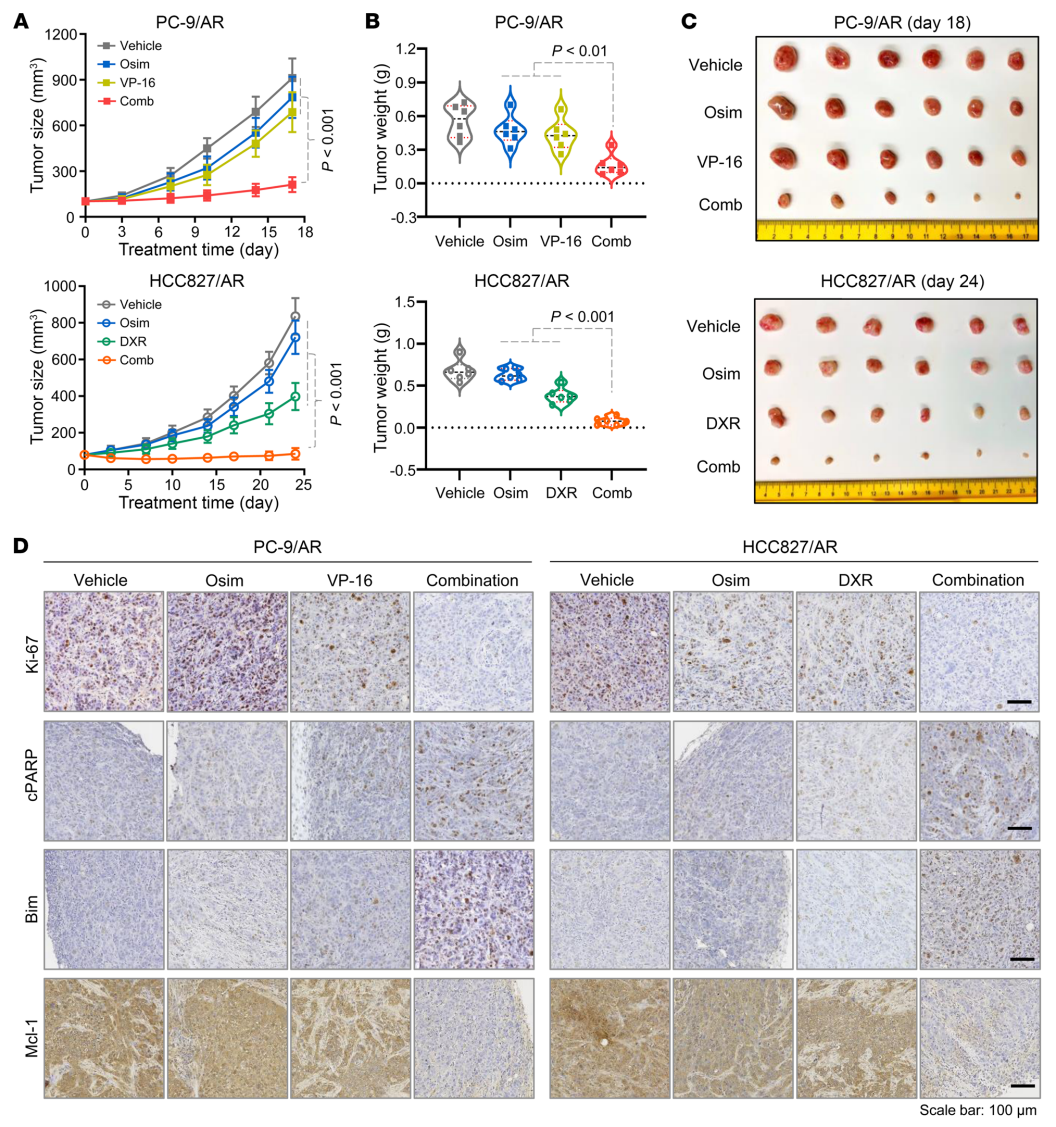
The combination of osimertinib with DXR or VP-16 effectively inhibits the growth of osimertinib-resistant EGFRm NSCLC xenografts in vivo, with modulation of several critical protein biomarkers in tumor tissues. PC-9/AR or HCC27/AR cells grown in nu/nu mice as xenograft tumors (*n* = 6/group) were treated with vehicle, osimertinib (Osim) alone (5 mg/kg, daily, oral gavage), DXR alone (1 mg/kg/d, daily, i.p.), VP-16 alone (1 mg/kg/d, daily, i.p.), or the indicated combinations. Tumor sizes were measured at the indicated time points (**A**). At the end of treatment, tumors in each group were also weighed (**B**) and photographed (**C**). The data in each group represent mean ± SEM of 6 tumors from 6 mice. The proteins of interest as indicated were stained with IHC. (**D**) Statistical differences among multiple groups were conducted with 1-way ANOVA test. Scale bar: 100 μm.

We next examined the effects of the combination of osimertinib with DXR and VP-16 on the growth of osimertinib-resistant xenografts in nude mice. As presented in Figure 2, the combination of osimertinib with either VP-16 or DXR was significantly more effective than either agent alone in inhibiting the growth of PC-9/AR or HCC827/AR tumors based on changes in both tumor size and weight (Figure 2, A–C). Therefore, it is clear that the combination of osimertinib and a Topo II inhibitor effectively inhibits the growth of osimertinib-resistant tumors in vivo. Mice receiving the combinations had body weight comparable to those treated with either single agent alone (Supplemental Figure 4), demonstrating that the combinations do not enhance toxicity in mice while potentiating therapeutic efficacy. We further detected the levels of Ki-67, a well-known cell proliferation marker; cleaved PARP (cPARP), a hallmark of apoptosis; and Bim and Mcl-1, 2 critical apoptosis-regulating proteins modulated by the combinations, as demonstrated above, in tumor tissues receiving these treatments. As shown in Figure 2D, the tissues from mice treated with both combinations displayed the fewest Ki-67–positive cells and the most cPARP-positive cells in comparison with other tissues receiving osimertinib, VP-16, or DXR treatment, further indicating that the combinations exert enhanced growth-inhibitory effects, with augmented induction of apoptosis against osimertinib-resistant tumors. In agreement with increased cPARP, the highest levels of Bim and lowest levels of Mcl-1 were detected in tissues receiving the combination treatment in comparison with tissues treated with each single agent (Figure 2D), validating our finding of the critical roles of Bim and Mcl-1 modulation in mediating enhanced apoptosis by the combination in vivo.

Moreover, we compared the effects of osimertinib combined with VP-16, cisplatin (CDDP), and paclitaxel (PTX; Taxol), respectively, on the growth of HCC827/AR tumors under the same conditions. While the combination of osimertinib with VP-16 effectively inhibited the growth of HCC827/AR tumors more significantly than either single agent alone, as demonstrated above, the combination of osimertinib with either CDDP or PTX failed to show enhanced effect on suppressing the growth of HCC827 tumors (Supplemental Figure 5). These data are consistent with our in vitro finding as presented above.

Considering that the above in vivo experiments were conducted in nude mice that are immunocompromised, we then examined the potential toxicities of osimertinib combined with VP-6 and DXR, respectively, with the same treatments used above in immunocompetent mice. After 5 weeks of treatments, the mouse body weights in the combination groups were comparable with those in the single-agent treatment groups (Supplemental Figure 6A). The histological examination of tissues from the major organs, including heart, liver, lung, kidney, and spleen, among the different groups did not show a difference (Supplemental Figure 6B). The detection of various serum proteins or enzymatic activities among the tested groups did not show a significant difference either (Supplemental Figure 6C). Therefore, the combinations are well-tolerated in the immunocompetent mice as well.

### Osimertinib decreases Topo IIα levels, resulting in induction of DNA damage and subsequent apoptosis in EGFRm NSCLC cell lines.

**Figure 3:**
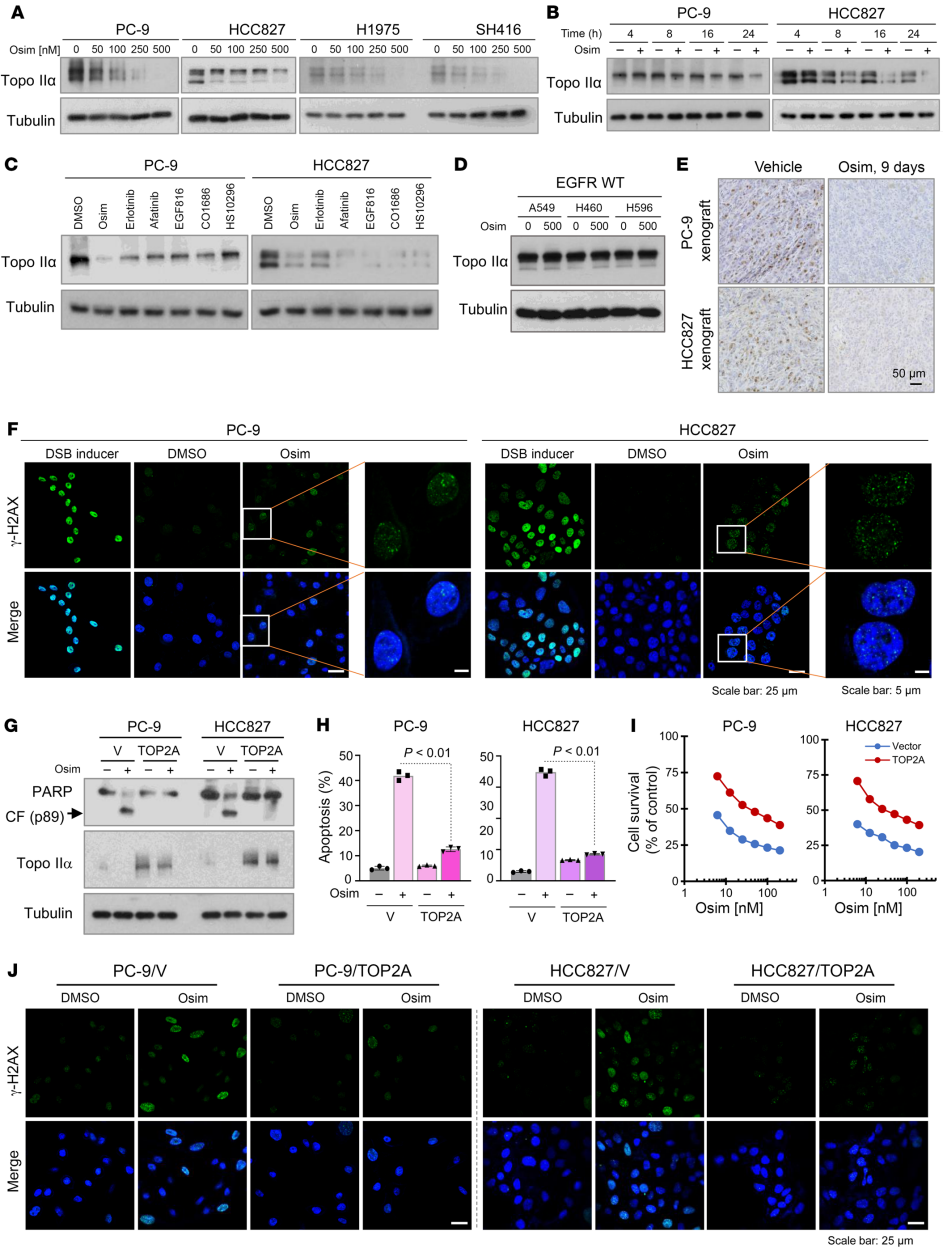
Osimertinib, as well as other EGFR-TKIs, decreases the levels of Topo IIα and induces γ-H2AX foci formation in EGFRm NSCLC cells and tissues, and overexpression of ectopic *TOP2A* attenuates the effects of osimertinib on induction of apoptosis, decreasing cell survival, and increasing γ-H2AX foci formation. (**A**–**D**) The given cell lines were exposed to varied concentrations of osimertinib (Osim) for 24 hours (**A**), 200 nM osimertinib for different times (**B**), 200 nM different EGFR-TKIs for 24 hours (**C**), or 500 nM osimertinib for 24 hours (**D**). Proteins of interest were detected with Western blotting. (**E**) Topo IIα in tissues was detected with IHC. Scale bar: 50 μm. (**F** and **J**) The indicated cell lines were exposed to 250 nM osimertinib for 16 hours and then stained with anti–γ-H2AX antibody and DAPI. DSB inducer here served as a positive control and was used at 100 μM for 1 hour of treatment. Scale bar: 25 μm (**F** and **J**); 5 μm (**F**, high-magnification images). (**G**–**I**) The indicated cell lines were exposed to DMSO or 200 nM osimertinib for 18 hours (**G**) or 24 hours (**H**) or treated with different concentrations of osimertinib for 3 days (**I**). The proteins of interest were detected with Western blotting (**G**), and apoptotic cells were detected with annexin V staining/flow cytometry (**H**). Each bar in **H** represents mean ± SD of triplicate treatments. Cell numbers were measured by the SRB assay and are expressed as mean ± SD of 4 replicate determinations (**I**). Statistical differences between 2 groups were conducted with 2-sided unpaired Student’s *t* test.

We were interested in the mechanisms underlying the activity of DXR and VP-16 in overcoming acquired resistance to osimertinib, as demonstrated above. One common property of DXR and VP-16 is that they are both Topo II inhibitors, making them distinct from other chemotherapeutic agents tested. Analysis of data from The Cancer Genome Atlas (TCGA) revealed that *TOPA2* expression was significantly higher in lung adenocarcinoma tissues than in normal tissues (Supplemental Figure 7A) and was significantly associated with reduced OS (Supplemental Figure 7B). In EGFRm lung adenocarcinoma, high *TOP2A* expression was also significantly associated with reduced OS (Supplemental Figure 7C). Therefore, we questioned whether Topo II inhibition plays a critical role in mediating therapeutic efficacy of osimertinib against EGFRm NSCLC or whether osimertinib exerts Topo II inhibitory activity, leading to DNA damage and subsequent apoptotic cell death in EGFRm NSCLC cells. We first examined the effect of osimertinib on modulating the levels of Topo II in different EGFRm NSCLC cell lines and found that osimertinib effectively decreased the levels of Topo IIα in a concentration-dependent manner in 4 different EGFRm NSCLC cell lines, PC-9, HCC827, H1975, and SH416 (Figure 3A). However, osimertinib, under the same tested condition, did not reduce the levels of Topo IIβ, another isoform of human Topo II (Supplemental Figure 8). The reduction of Topo IIα occurred early, at 8 hours, and was sustained for 24 hours in both PC-9 and HCC827 cell lines (Figure 3B). Furthermore, other EGFR-TKIs, including erlotinib (first generation), afatinib (second generation), EGF816 (third generation), CO1686 (third generation), and HS-10296 (third generation), also decreased Topo IIα levels in these cell lines (Figure 3C). As expected, even at 500 nM osimertinib did not decrease Topo IIα levels in NSCLC cell lines with WT *EGFR* gene (Figure 3D). In both PC-9 and HCC827 xenograft tumor tissues treated with osimertinib for 9 days, Topo IIα levels were clearly decreased in comparison with corresponding control tissues exposed to vehicle (Figure 3E). These results demonstrate that osimertinib as well other EGFR-TKIs decrease Topo IIα levels in EGFRm NSCLC cells and tissues.

We next determined whether osimertinib-induced Topo IIα is accompanied by enhanced DNA DSBs or damage by detecting γ-H2AX foci formation, a classical assay for detection of DNA DSBs (11, 12). In both PC-9 and HCC827 cells, we detected cells positive for γ-H2AX foci staining, which were hardly detected in DMSO-treated control cells (Figure 3F). Therefore, it is clear that osimertinib induces DNA damage in EGFRm NSCLC cells.

We further determined whether Topo IIα reduction is a critical event contributing to induction of DNA damage and apoptosis by osimertinib in EGFRm NSCLC. To this end, we enforced overexpression of an ectopic *TOP2A* gene in both PC-9 and HCC827 cells and then checked their responses to osimertinib in terms of DNA damage, apoptosis induction, and cell survival. The results show that osimertinib effectively induced PARP cleavage, increased annexin V–positive cells, and decreased cell survival in vector control cells but had reduced effects in *TOP2A*-expressing cells (Figure 3, G–I). Consistently, osimertinib increased γ-H2AX foci staining in vector control cells but only weakly in *TOP2A*-expressing cells (Figure 3J). These data clearly demonstrate that enforced overexpression of the ectopic *TOP2A* gene in EGFRm NSCLC cells attenuates the ability of osimertinib to induce DNA damage and apoptosis and to decrease cell survival, suggesting an essential role of Topo II inhibition in mediating therapeutic efficacy of osimertinib against EGFRm NSCLC cells.

### Osimertinib decreases Topo IIα levels via promoting GSK3-dependent and FBXW7-mediated proteasomal degradation and suppressing SMURF2 expression in EGFRm NSCLC cell lines.

**Figure 4:**
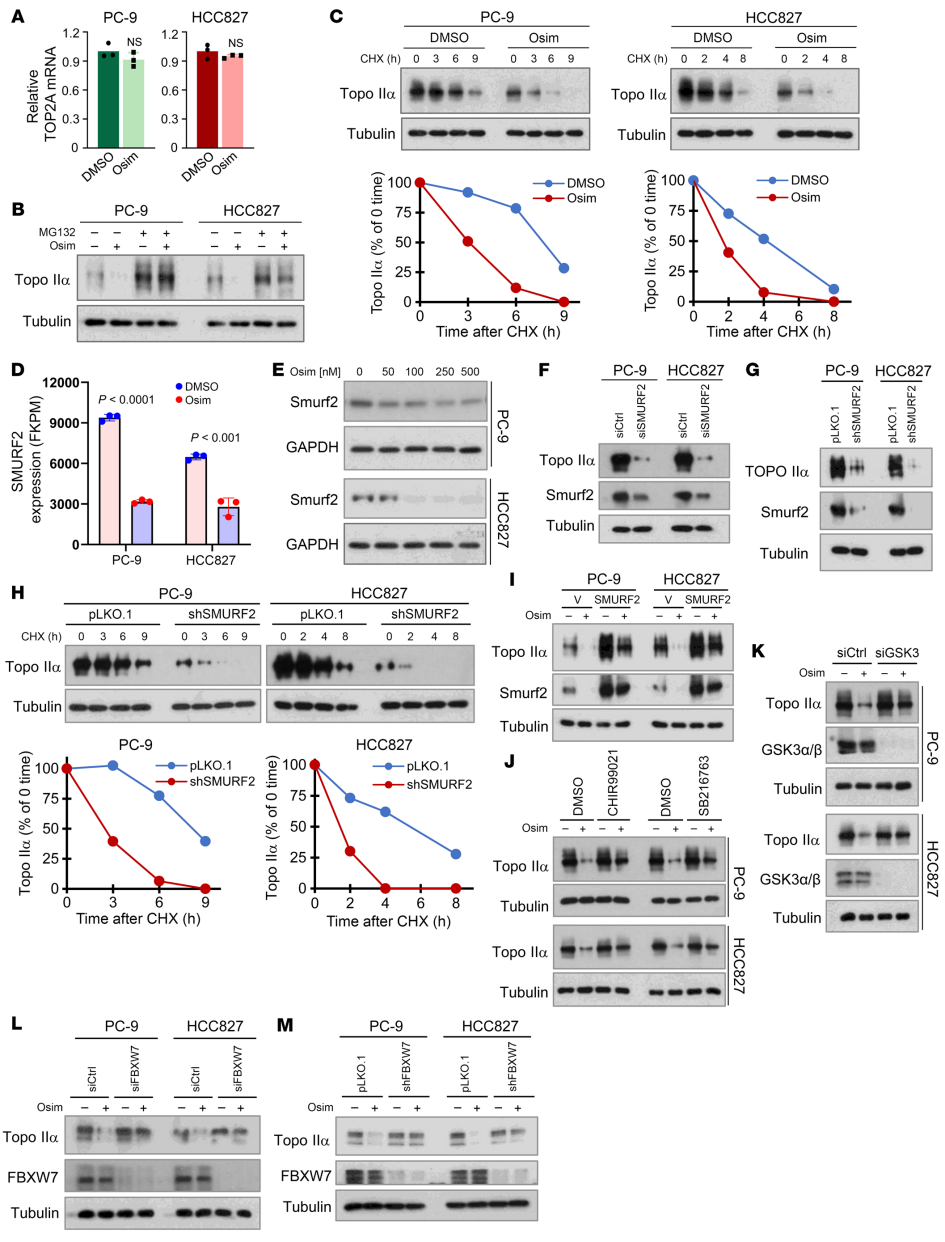
Osimertinib does not affect *TOP2A* transcription but promotes GSK3-dependent and FBXW7-mediated Topo IIα protein degradation associated with suppression of *SMURF2* expression. (**A**) The tested cell lines were exposed to 200 nM osimertinib (Osim) for 16 hours. *TOP2A* mRNA was detected with quantitative reverse transcription PCR. NS, not significant with 2-sided unpaired Student’s *t* test. (**B**) The tested cell lines were pretreated with 10 μM MG132 for 30 minutes and then cotreated with DMSO or 200 nM osimertinib for another 6 hours. (**C**) Both PC-9 and HCC827 cells were treated with 200 nM osimertinib for 16 hours followed by the addition of 10 μg/mL CHX and then harvested at the indicated times. (**D**) RNA-Seq detection of *SMURF2* mRNA expression in the given cell lines exposed to 100 nM osimertinib for 14 hours. (**E**) The tested cell lines were exposed to varied concentrations of osimertinib as indicated for 24 hours. (**F** and **G**) The tested cell lines were transfected with the given siRNAs or infected with lentiviruses carrying the given shRNA for 48 hours. (**H**) The tested cell lines were exposed to 10 μg/mL CHX and then harvested at different times as indicated. (**I**) The tested cell lines were treated with 200 nM osimertinib for 24 hours. (**J**) Both PC-9 and HCC827 were pretreated with 10 μM CHIR99021 or SB216763 for 30 minutes and then cotreated with 200 nM osimertinib for an additional 16 hours. (**K** and **L**) The tested cell lines were transfected with scrambled GSK3 (**K**) or FBXW7 (**L**) siRNA for 48 hours followed by treatment with 200 nM osimertinib for another 24 hours. (**M**) The indicated cell lines expressing pLKO.1 or shFBXW7 were exposed to 200 nM osimertinib for 24 hours. The proteins with the aforementioned treatments were detected with Western blotting. Band intensities were quantified with ImageJ (NIH) software and plotted as percentage of 0 time (**C** and **H**).

To understand the molecular mechanism by which osimertinib decreases Topo IIα levels in EGFRm NSCLC cells, we studied mRNA levels and found that osimertinib did not alter *TOP2A* mRNA levels (Figure 4A), suggesting a possible posttranscriptional mechanism. Considering that Topo IIα levels are modulated by a posttranslational mechanism (13), we then determined the effect of proteasome inhibition on Topo IIα reduction induced by osimertinib and found that the presence of the proteasome inhibitor, MG132, abolished the ability of osimertinib to decrease Topo IIα levels in both PC-9 and HCC827 cell lines (Figure 4B). Moreover, Topo IIα was degraded much faster in PC-9 and HCC827 cells exposed to osimertinib than in DMSO-treated cells, as demonstrated by the cycloheximide (CHX) chase assay (Figure 4C). Altogether, we concluded that osimertinib facilitates proteasomal degradation of Topo IIα, leading to Topo IIα reduction in EGFRm NSCLC cells.

Smurf2 is a protein E3 ubiquitin ligase that is involved in negative regulation of Topo IIα degradation via modifying Topo IIα ubiquitination status by reducing degradation-promoting K48 polyubiquitination and increasing monoubiquitination (14). We noted that *SMURF2* gene expression was significantly decreased in both PC-9 and HCC827 cells, as revealed in our RNA-Seq analysis (Figure 4D). Smurf2 protein reduction in EGFRm NSCLC cells exposed to osimertinib was also confirmed with Western blotting (Figure 4E). The presence of MG132 did not rescue Smurf2 reduction caused by osimertinib (Supplemental Figure 9), supporting its modulation by osimertinib at a transcriptional level. To determine whether there is a connection between Smurf2 suppression and Topo IIα degradation, we knocked down *SMURF2* gene expression with either siRNA or shRNA in EGFRm NSCLC cells and then examined Topo IIα alterations in these cell lines. Topo IIα levels were decreased in cells transfected with *SMURF2* siRNA or infected with *SMURF2* shRNA lentiviruses (Figure 4, F and G). Moreover, knockdown of the *SMURF2* gene in both PC-9 and HCC827 cell lines facilitated the degradation of Topo IIα in the CHX chase assay (Figure 4H). In contrast, enforced overexpression of an ectopic *SMURF2* gene in these cell lines elevated basal levels of Topo IIα and attenuated the ability of osimertinib to decrease Topo IIα levels (Figure 4I). These data strongly suggest that Smurf2 downregulation is tightly linked to Topo IIα degradation in EGFRm NSCLC cells exposed to osimertinib.

To identify the actual E3 ubiquitin ligase that mediates Topo IIα polyubiquitination and subsequent proteasomal degradation we focused on FBXW7, because Topo IIα was demonstrated to undergo GSK3-dependent and FBXW7-mediated proteasomal degradation (15), whereas osimertinib induces GSK3-dependent and FBXW7-mediated degradation of SREBP1 in EGFRm NSCLC cells, as we recently demonstrated (16). In the presence of the GSK3 inhibitor, either CHIR99021 or SB216763, osimertinib had diminished effects on reducing Topo IIα levels in both PC-9 and HCC827 cells (Figure 4J). Similarly, knockdown of GSK3 in these cell lines rescued Topo IIα reduction induced by osimertinib (Figure 4K). Consistently, knockdown of FBXW7 with either siRNA or shRNA in these cell lines prevented osimertinib-induced Topo IIα reduction as well (Figure 4, L and M). Collectively, these results clearly demonstrate that osimertinib induces GSK3-dependent and FBXW7-mediated proteasomal degradation of Topo IIα.

### Topo IIα levels are elevated in osimertinib-resistant cell lines and in tissues from patients with EGFRm NSCLC who relapsed from EGFR-TKI treatment.

**Figure 5:**
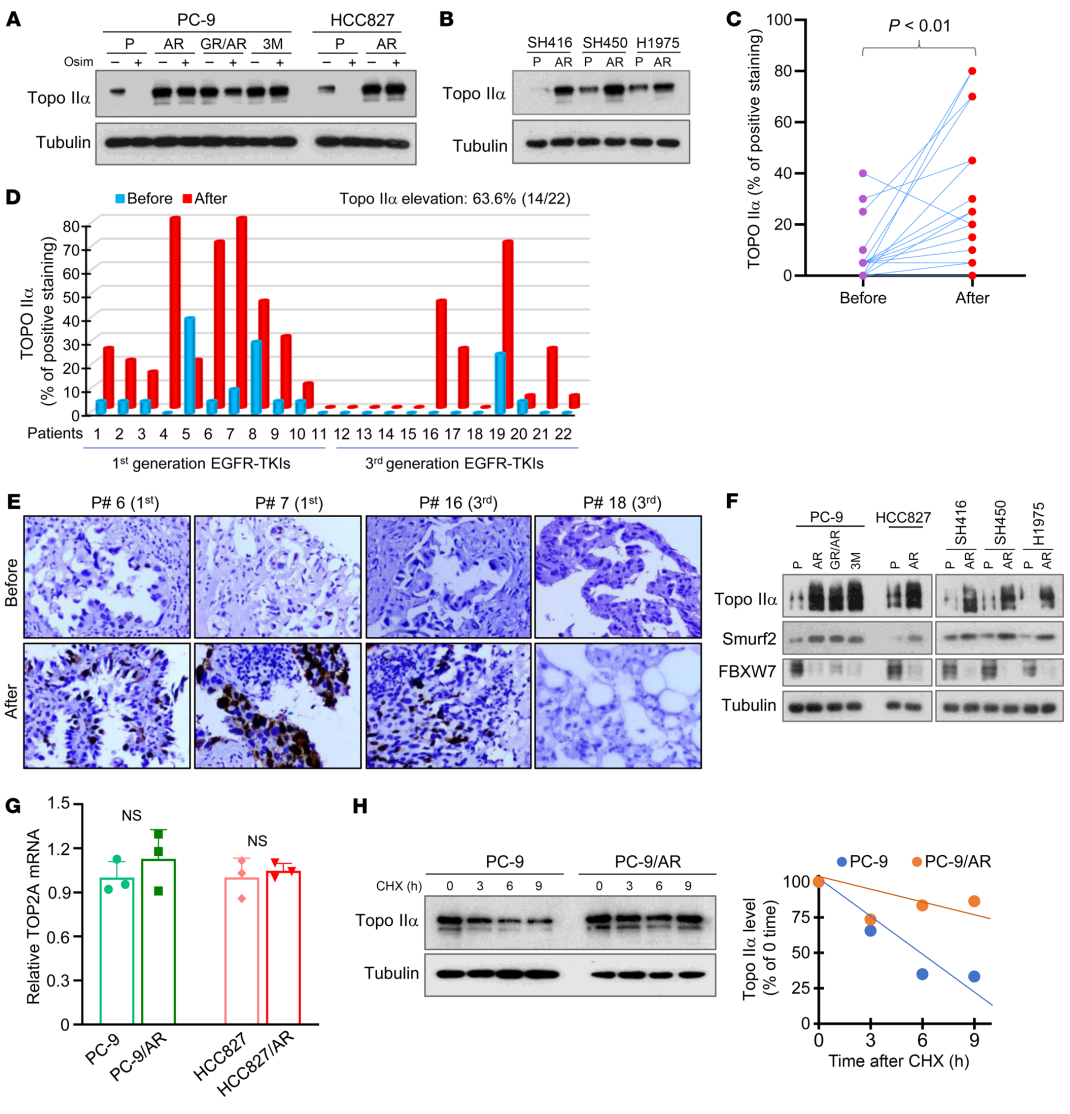
Topo IIα levels are elevated in EGFRm NSCLC cell lines with acquired resistance to osimertinib and tissue samples from patients with EGFRm NSCLC relapsed from EGFR-TKI treatment, which are resistant to osimertinib modulation and have elevated Smurf2 and decreased FBXW7, unchanged mRNA expression, and increased stability of Topo IIα. (**A**, **B**, and **F**) Whole-cell protein lysates were prepared from the given osimertinib-resistant cell lines exposed to 1,000 nM osimertinib (Osim) for 24 hours (**A**) or from untreated given cell lines with similar densities (**B** and **F**). The indicated proteins were detected with Western blotting. (**C**–**E**) Topo IIα in human EGFRm NSCLC tissues before and after relapse from treatment using EGFR-TKIs, including osimertinib, was stained with IHC. Statistical analysis was conducted with 2-sided paired Student’s *t* test. Original magnification, ×20. (**G**) *TOP2A* mRNA expression in the indicated cell lines were detected with quantitative reverse transcription PCR. NS, not significant with 2-sided unpaired Student’s *t* test. (**H**) The tested cell lines were exposed to 10 μg/mL CHX and then harvested at different times as indicated for subsequent Western blotting. Band intensities were quantified with ImageJ (NIH) software and plotted as percentage of 0 time.

To further elucidate the critical role of Topo IIα in mediating the response of NSCLC cells to osimertinib or EGFR-TKIs, we compared basal levels of Topo IIα between osimertinib-resistant cell lines and their matched parental cell lines and found that Topo IIα levels were in general higher in the tested osimertinib-resistant cell lines than in their corresponding parental cell lines (Figure 5, A and B). Osimertinib treatment had little or no effect on decreasing Topo IIα levels in these resistant cell lines, in contrast to its effects on Topo IIα in the parental cell lines (Figure 5A). We also detected Topo IIα in paired tissues from patients with EGFRm NSCLC receiving EGFR-TKI treatment, including osimertinib, and found that Topo IIα levels were in general significantly elevated after relapse from the treatment compared with their baseline levels (Figure 5C). Specifically, we detected Topo IIα elevation in 64% (14 of 22) of relapsed EGFRm NSCLC tissues (Figure 5D), with strong positive staining in some cases (e.g., patients 6, 7, and 16; Figure 5E). Therefore, Topo IIα levels are elevated not only in osimertinib-resistant EGFRm NSCLC cell lines but also in EGFRm NSCLC tissues relapsed from EGFR-TKI treatment.

To have an insight into the mechanism of elevated Topo IIα in osimertinib-resistant cell lines, we detected the levels of Smurf and FBXW7 in EGFRm NSCLC cell lines and their derived osimertinib-resistant cell lines. In a similar way to Topo IIα, Smurf levels were elevated in the osimertinib-resistant cell lines. In contrast, FBXW7 levels were lower in these resistant cell lines than their matched parental cell lines (Figure 5F). We also compared *TOP2A* mRNA levels between osimertinib-resistant cell lines and their matched parental cell lines and found that *TOP2A* mRNA expression was not significantly elevated in the osimertinib-resistant cell lines (Figure 5G). Finally, we conducted the CHX assay to compare the stabilities of Topo IIα between PC-9 and PC9/AR cell lines and found that Topo IIα was degraded much faster in PC-9 cells than in PC-9/AR cells (Figure 5H), suggesting increased stability of Topo IIα in PC-9/AR cells. These results together strongly suggest that Topo IIα elevation in the osimertinib-resistant cell lines is likely due to the alterations of Smurf and FBXW7 that lead to stabilization of Topo IIα.

### Genetic knockdown of TOP2A in osimertinib-resistant cell lines restores cell responses to osimertinib in inducing DNA damage and apoptosis.

**Figure 6:**
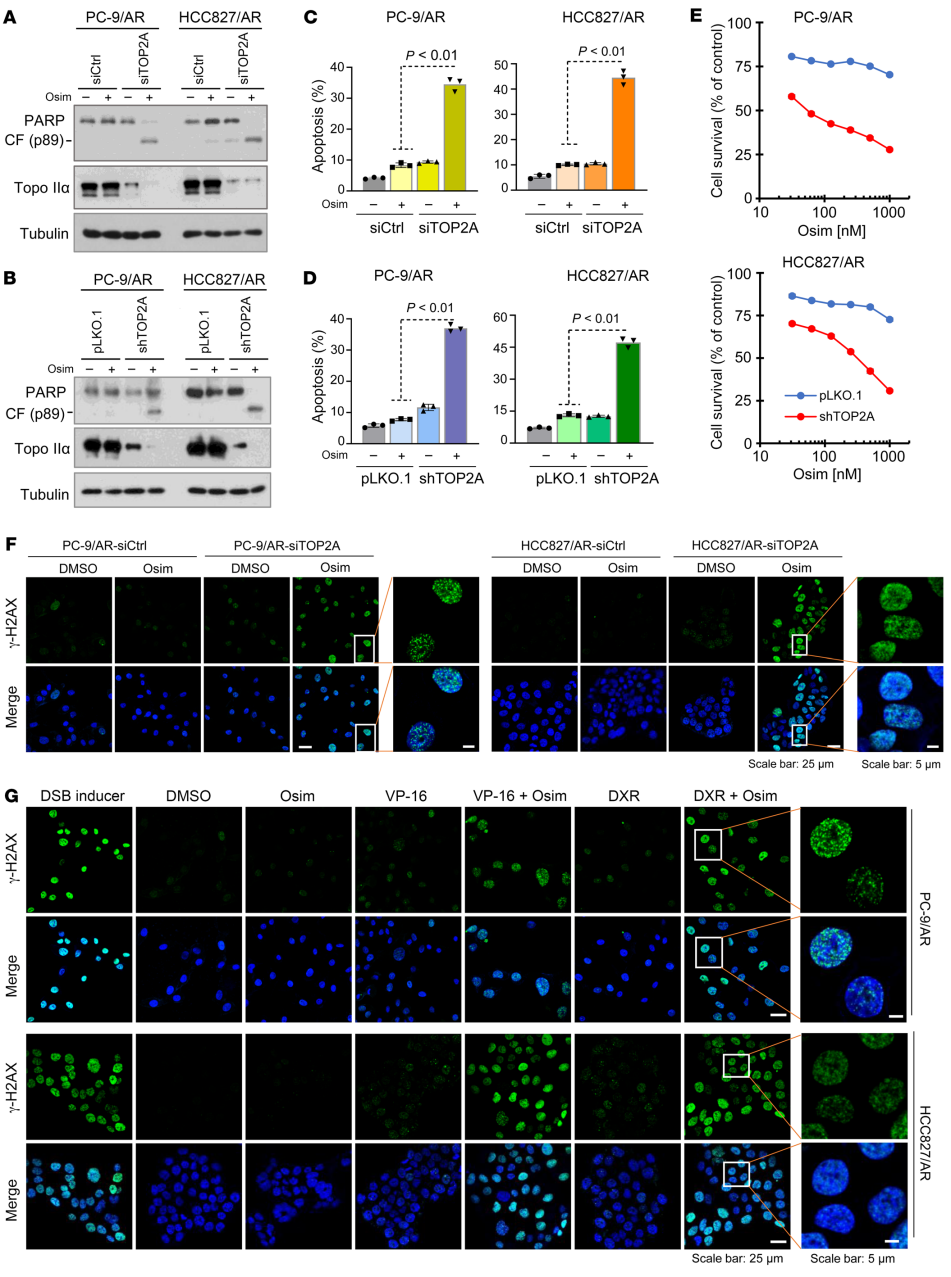
Genetic knockdown of *TOP2A* expression in osimertinib-resistant cells restores their response to osimertinib in inducing apoptosis, decreasing cell survival, and increasing DNA damage, similar to the effect of combined osimertinib and Topo II inhibitor on enhancing induction of DNA damage in these resistant cell lines. (**A**–**E**) PC-9/AR and HCC827/AR cells transfected with scrambled control or *TOP2A* siRNA for 48 hours (**A** and **C**) or expressing pLKO.1 or shTOP2A (**B**, **D**, and **E**) were exposed to 200 nM osimertinib for 24 hours (**A** and **B**), 48 hours (**C** and **D**), or 72 hours (**E**). Topo IIα and PARP cleavage were detected with Western blotting (**A** and **B**). Annexin V–positive cells were determined with flow cytometry (**C** and **D**). Cell numbers were estimated with the SRB assay (**E**). The data represent mean ± SD of triplicate (**C** and **D**) or 4 replicate (**E**) determinations. Statistical analysis was conducted with 2-sided unpaired Student’s *t* test. CF, cleaved form. (**F**) The indicated cell lines were transfected with scrambled control or *TOP2A* siRNA for 48 hours and then exposed to 250 nM osimertinib for an additional 24 hours. The cells were then subjected to detection of γ-H2AX foci using IF staining with anti–γ-H2AX antibody. (**G**) The indicated cell lines were treated with DMSO, 250 nM osimertinib, 100 nM (PC-9/AR) or 250 nM (HCC827/AR) DXR, 1 μM VP-16, or their respective combinations as indicated for 24 hours and then subjected to detection of γ-H2AX foci using IF staining with anti–γ-H2AX antibody. DSB (100 μM for 1 hour) here was used as a positive control. Scale bar: 25 μm (**F** and **G**); 5 μm (**F** and **G**, high-magnification images).

If elevated Topo IIα plays a critical role in conferring resistance to osimertinib, we speculated that enforced suppression of *TOP2A* expression by gene knockdown in osimertinib-resistant cell lines should restore their sensitivity to osimertinib. As presented in Figure 6, knockdown of *TOP2A* with either siRNA or shRNA in both PC-9/AR and HCC827/AR cell lines sensitized the cells to undergo apoptosis, as demonstrated by increased PARP cleavage (Figure 6, A and B) and annexin V–positive cells (Figure 6, C and D). Consistently, osimertinib was much more active in decreasing the survival of both PC-9/AR and HCC827/AR cells expressing shTOP2A, while it did so only minimally in their corresponding control cells (Figure 6E), further demonstrating the effect of Topo IIα suppression on sensitizing osimertinib-resistant cells to osimertinib. Moreover, osimertinib failed to induce γ-H2AX foci–positive cells in the control PC-9/AR and HCC827/AR cell lines, but clearly increased the number of cells with positive nuclear γ-H2AX foci staining in cells transfected with *TOP2A* siRNA (Figure 6F). Therefore, enforced inhibition of Topo IIα with gene knockdown in osimertinib-resistant cells restores their response to osimertinib in inducing DNA damage, increasing apoptosis and decreasing cell survival.

### Osimertinib in combination with a Topo II inhibitor enhances DNA damage in osimertinib-resistant cells.

As demonstrated above, induction of DNA damage is a critical event for osimertinib to exert its therapeutic activity, which is lost in osimertinib-resistant cell lines and can be restored by genetic knockdown of *TOP2A* expression. Therefore, we further determined whether the combination of osimertinib with a Topo II inhibitor leads to enhanced DNA damage in osimertinib-resistant cells. By performing γ-H2AX foci staining, we found that the combination of osimertinib with either DXR or VP-16 effectively enhanced the number of γ-H2AX foci–positive cells, whereas either of the tested agents alone caused little or no increase in γ-H2AX foci–positive cells (Figure 6G). This finding indicates that the combination of osimertinib with a Topo II inhibitor indeed enhances DNA damage in osimertinib-resistant cells, in agreement with their enhanced induction of apoptosis as demonstrated above.

### The combination of osimertinib and VP-16 exerts augmented therapeutic efficacy against the growth of EGFRm NSCLC patient-derived xenografts and delays emergence of acquired resistance to osimertinib.

**Figure 7:**
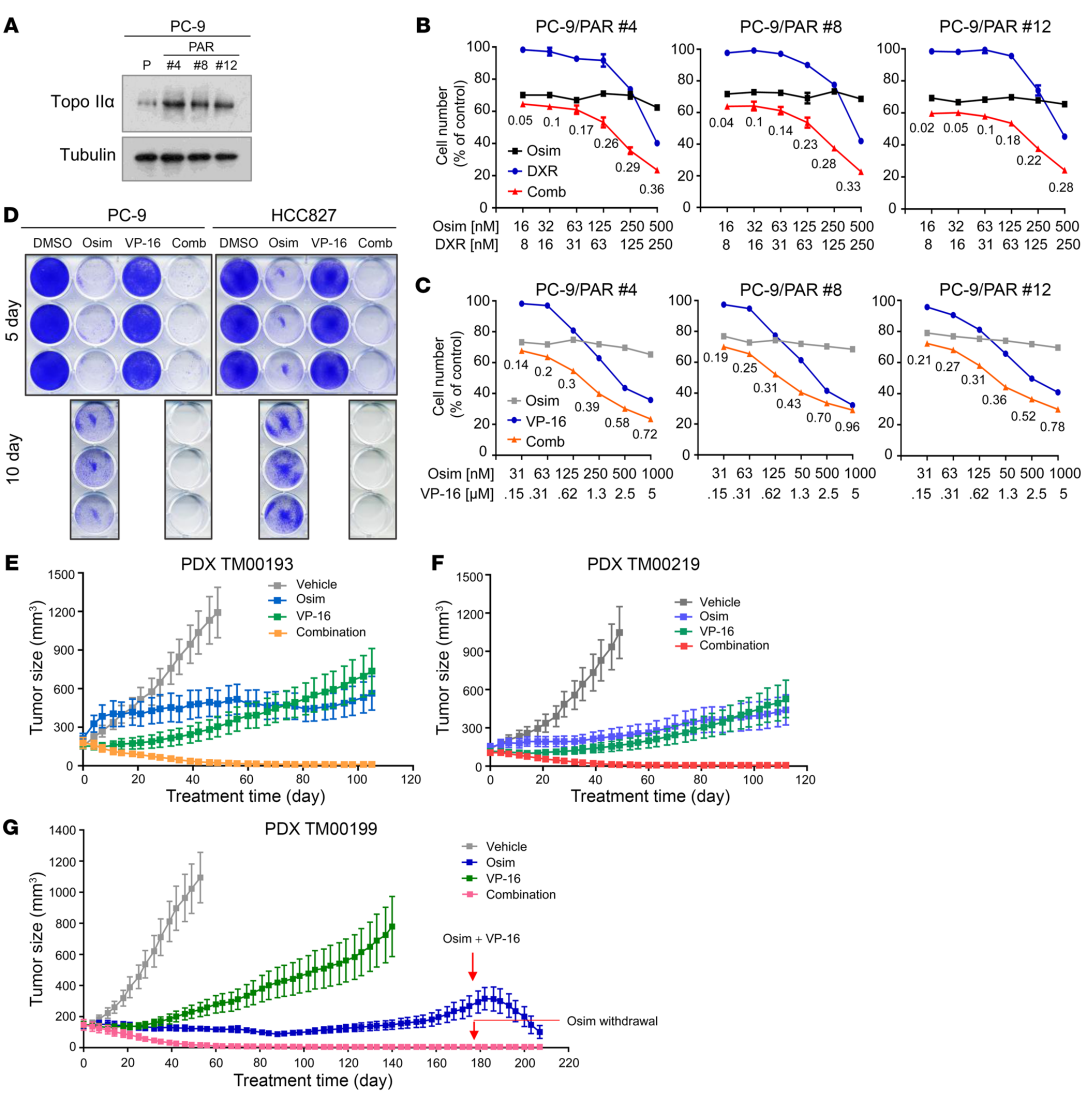
Osimertinib combined with a Topo II inhibitor synergistically decreases the survival of EGFRm NSCLC cell lines with primary resistance to osimertinib expressing elevated levels of Topo IIα, eliminates DTCs, and regresses different EGFRm PDX tumors in vivo with long-term remissions. (**A**) Detection of basal levels of Topo IIα in the indicated cell lines with Western blotting. (**B** and **C**) The given cell lines were exposed to varied concentrations of osimertinib (Osim), DXR, or VP-16 alone as indicated and the combination of osimertinib with DXR or VP-16. After 3 days, cell numbers were determined with the SRB assay. The data represent mean ± SD of 4 replicate determinations. (**D**) The indicated cell lines seeded in 12-well plates were treated with 50 nM osimertinib, 150 nM VP-16, or a combination; these treatments were repeated with fresh medium every 2 days. After 5 or 10 days, the cells were fixed, stained with crystal violet dye, and images were taken. (**E**–**G**) The indicated PDXs in nude mice (6 tumors/group) were treated with vehicle, 5 mg/kg osimertinib (daily, oral gavage), 1 mg/kg VP-16 (daily, i.p.), or the combination of osimertinib and VP-16. The data represent mean ± SEM of 6 tumors.

Beyond the demonstrated effects of osimertinib combined with a Topo II inhibitor in EGFRm NSCLC cell lines with acquired resistance to osimertinib, we also examined the effects of these combinations on the growth of a few cell lines with primary resistance to osimertinib isolated from sensitive EGFRm PC-9 cells. The rationale was based on our finding that several PC-9–derived cell lines with primary resistance to osimertinib (17) had elevated levels of Topo IIα (Figure 7A). Similar to what we found in EGFRm NSCLC cell lines with acquired resistance to osimertinib above, the combination of osimertinib with DXR or VP-16 was more effective than either single agent alone in decreasing the survival of the 3 cell lines with primary osimertinib resistance with CIs of less than 1 (Figure 7, B and C), indicating synergistic effects. Beyond the presence of primarily resistant clones in sensitive EGFRm NSCLC cell populations as a key mechanism accounting for the emergence of acquired resistance to EGFR-TKIs, drug-tolerant persister cells (DTCs) surviving in the initial period of treatment represent another critical origin accounting for the emergence of acquired resistance (18, 19). We clearly detected DTCs in cells treated with osimertinib alone after a 10-day treatment but not in cells exposed to the combination of osimertinib and VP-16 (Figure 7D), indicating that the combination is also effective in eliminating DTCs. Because of these promising findings, we then reasonably asked whether the combination of osimertinib with a Topo II inhibitor such as VP-16 potentiates the growth suppression of EGFRm NSCLC tumors and delays emergence of acquired resistance to osimertinib in vivo. The 3 EGFRm NSCLC patient-derived xenografts (PDXs), TM00193, TM00219, and, particularly, TM00190, were all responsive to osimertinib treatment, albeit with varied sensitivities. Among them, TM00193 was relatively less responsive to osimertinib. It did not respond well to osimertinib in the initial period of treatment but responded after 20 days, although the growth was retarded (Figure 7E and Supplemental Figure 10A). TM00219 and particularly TM00190 were relatively sensitive to osimertinib, but did grow back as the treatment times were prolonged (Figure 7, F and G, and Supplemental Figure 10, B and C), indicating emergence of acquired resistance. All 3 PDXs were sensitive to VP-16 in the initial period of treatment (e.g., up to 40 days) under the tested conditions and then gradually became less responsive to treatment (Figure 7, E–G, and Supplemental Figure 10), suggesting the development of resistance as well. However, the combination of osimertinib and VP-16 in each model quickly reduced the sizes of tumors even after a few days of treatment. After about 40 days, the tumors were all reduced to minimal sizes; these effects were maintained for over 100 days of treatment (Figure 7, E–G, and Supplemental Figure 10). In the TM00190 model, the tumor-suppressive effect of the combination was maintained more than 170 days while tumors treated with osimertinib alone had started to resume growth (Figure 7G and Supplemental Figure 10C). We did not observe regrowth of tumors treated with the combination of osimertinib and VP-16, even after 1 month of withdrawing the treatment, indicating no relapse. When relapsed tumors treated with osimertinib alone were switched to the combination of osimertinib and VP-16 after day 170, tumor shrinkage was observed (Figure 7G and Supplemental Figure 10C), further confirming the efficacy of the combination in overcoming acquired resistance to osimertinib in this PDX model. These results convincingly demonstrate that the inclusion of VP-16 in EGFR-targeted therapy (e.g., with osimertinib) may lead to augmented therapeutic efficacy and delay or even prevent the emergence of acquired resistance. In this study with different EGFRm NSCLC PDXs, mice treated with the combination of osimertinib and VP-16 had comparable body weights to those treated with osimertinib alone even after over 200 days (Supplemental Figure 11), indicating the favorable tolerability of the combination while augmenting therapeutic efficacy against the growth of EGFRm NSCLC tumors.

## Discussion

Platinum-based chemotherapy is standard of care following development of resistance to osimertinib in the clinic. An open-label randomized phase II study that evaluated osimertinib combined with carboplatin-pemetrexed in comparison with osimertinib monotherapy in patients with EGFRm NSCLC who experienced disease progression associated with the emergence of EGFR T790M resistance mutation during first-line EGFR-TKI therapy failed to demonstrate prolongation of progression-free survival (20). Other clinical trials with osimertinib plus platinum-pemetrexed in newly diagnosed EGFRm advanced/metastatic NSCLC showed a manageable safety with tolerability profile (21) and significantly prolonged PSF compared with osimertinib alone (22). Nonetheless, the rationale or molecular mechanism for the combination is unclear.

In our study, the combination of osimertinib with CDDP, carboplatin, PTX, gemcitabine, fluorouracil (5-FU), cyclophosphamide, capecitabine, or vincristine did not show enhanced effects on decreasing the survival of osimertinib-resistant cell lines. Consistently, the combinations of osimertinib with CDDP and PTX, respectively, did not show enhanced effects on the suppression of osimertinib-resistant tumors in vivo either. Interestingly, DXR and VP-16, both of which share the common action of mechanism, Topo II inhibition, synergized with osimertinib to decrease the survival of osimertinib-resistant cell lines and to suppress the growth of osimertinib-resistant xenograft tumors, including PDXs, in vivo, thus generating interest in this combination. Efforts to demonstrate the underlying biology or scientific rationale in this study have led us to find a role of Topo IIα modulation in critically mediating the response of EGFRm NSCLC cells to osimertinib and likely other EGFR-TKIs based on the following findings: (a) osimertinib as well as other EGFR-TKIs effectively decreased the levels of Topo IIα via facilitating its proteasomal degradation; this process involves suppression of *SMURF* expression in the sensitive EGFRm NSCLC cell lines and tumors accompanied by induction of DNA damage; (b) enforced overexpression of the ectopic *TOP2A* gene attenuated the ability of osimertinib to induce DNA damage and apoptosis, conferring resistance to osimertinib; (c) Topo IIα levels were elevated in osimertinib-resistant cell lines, likely due to increased protein stability caused by Smurf2 elevation and FBXW7 reduction, and in the majority of EGFRm NSCLC tissue relapsed from EGFR-TKI treatment and were resistant to osimertinib modulation; and (d) enforced reduction of Topo IIα levels via gene knockdown restored the capacities of osimertinib-resistant cell lines to undergo DNA damage and apoptosis upon osimertinib treatment. Collectively, the current study has demonstrated an essential role of Topo IIα inhibition in mediating the therapeutic efficacy of osimertinib against EGFRm NSCLC cells and the scientific rationale for targeting Topo II to overcome acquired resistance to osimertinib. We realized the general limitation of gene overexpression strategy that often results in supraphysiologic levels of a tested protein. Under the specific scenario that Topo IIα levels were highly elevated in osimertinib-resistant EGFRm NSCLC cell lines, enforced expression of ectopic *TOP2A* gene in the sensitive EGFRm NSCLC cell lines with low levels of Topo IIα may mimic the situation in the resistant cell lines. Outcomes from this study should provide complementary support to *TOP2A* knockdown results for demonstrating the critical role of Topo II inhibition in mediating the therapeutic efficacy of osimertinib in EGFRm NSCLC cells.

Both DXR and VP-16 have long been used for the treatment of cancers, although they are used less frequently in NSCLC. Our findings thus warrant the clinical validation of this therapeutic strategy to overcome acquired resistance to osimertinib. It is well known that human cells express both Topo IIα and Topo IIβ enzymes (23, 24). Topo IIβ was previously identified as a major cellular target for DXR-induced cardiotoxicity (25). In this study, osimertinib at concentrations of up to 500 nM did not reduce the levels of Topo IIβ, indicating its selectivity in targeting Topo IIα. In our in vivo animal study, the combination of osimertinib with either DXR or VP-16 was well tolerated in nude mice without apparently increasing toxicity (i.e., body weight loss). In agreement, the treatment of regular immunocompetent mice with the combinations did not show the increased toxicity either, further indicating their safety. One limitation of the study is that the majority of the mechanistic findings were primarily generated from PC-9 and HCC827 cell lines, although they are widely used in the community. This is largely due to the limited availability of characterized EGFRm cell lines that are sensitive to EGFR-TKIs.

One important finding is that Topo IIα levels were elevated in several EGFRm NSCLC cell lines with acquired resistance to osimertinib, which was confirmed in over 60% of tissues from patients with EGFRm NSCLC who relapsed following treatment with EGFR-TKIs, including osimertinib, constituting a critical foundation for targeting Top o II to overcome acquired resistance to osimertinib and possibly other EGFR-TKIs. It is very likely that this strategy may work well in relapsed NSCLC with elevated Topo IIα expression. Therefore, Topo IIα elevation may be used as a predictive biomarker for selecting patients with disease relapse from osimertinib treatment to receive this therapeutic strategy, i.e., the combination of osimertinib with a Topo II inhibitor such as VP-16.

Instead of passively waiting until the development of acquired resistance, another sound and active strategy for managing the acquired resistance is to delay or prevent the inevitable emergence of acquired resistance through an early preventive intervention before disease progression, which can be achieved by using effective and tolerable combination regimens that interfere with the process of developing acquired resistance (19). VP-16 has long been used for the treatment of cancers, although it is used less frequently for NSCLC. Therefore, its safety profile in patients is well known to oncologists and expected to be manageable. In this study, the combination of osimertinib and VP-16 potentiated the growth suppression of 3 different EGFRm NSCLC PDXs compared with the efficacy of each single agent. Tumors receiving the combination treatment regressed to minimal or undetectable sizes without relapse over a long treatment period of more than 100 days. Strikingly, tumors remained suppressed with no sign of regrowth after withdrawal of the combination treatment for over 30 days. Beyond this promising efficacy, we did not observe enhanced toxicity in mice, even up to 200 days, indicating that the combination treatment is well tolerated in mice. The promising effect of this combination on delaying or preventing the emergence of acquired resistance is largely due to its effectiveness in eliminating both primarily resistant clones and DTCs present in the EGFRm NSCLC cell population, which both constitute a key mechanism accounting for the emergence of acquired resistance to EGFR-TKIs, including osimertinib (18, 19). Therefore, our findings in this regard warrant the clinical evaluation of the osimertinib and VP-16 combination in patients with EGFRm NSCLC as an effective regimen to delay or prevent the emergence of acquired resistance and prolong patient survival. VP-16 has been primarily used in the treatment of SCLC (26, 27). One interesting mechanism accounting for the development of acquired resistance to osimertinib is the transformation of SCLC (4, 28). This provides additional rationale for using VP-16 early in combination with osimertinib to delay or prevent the emergence of acquired resistance to osimertinib.

Smurf2 is a HECT-type E3 ubiquitin ligase and functions as a physiologic regulator of Topo IIα levels through physical interaction with Topo IIα and modification of its ubiquitination status by reducing degradation-promoting K48 polyubiquitination and increasing monoubiquitination that protects Topo IIα from proteasomal degradation (14). Indeed, we have demonstrated that suppression of *SMURF2* expression is tightly involved in the regulation of osimertinib-induced Topo IIα degradation because enforced overexpression of the ectopic *SMURF2* gene in EGFRm NSCLC cells elevated basal levels of Topo IIα and attenuated the ability of osimertinib to decrease Topo IIα levels, while knockdown of *SMURF2* expression resulted in facilitation of Topo IIα degradation and substantial reduction of Topo IIα levels in these cell lines. However, Smurf2 is not the E3 ubiquitin ligase responsible for the polyubiquitination and degradation of Topo IIα induced by osimertinib. In this study, we further suggest that osimertinib induces proteasomal degradation of Topo IIα, likely via a GSK3-dependent and FBXW7-mediated mechanism, in EGFRm NSCLC cells, since inhibition of either GSK3 or FBXW7 rescued Topo IIα reduction induced by osimertinib. This is consistent with our previous finding that osimertinib promotes GSK3-dependent and FBXW-mediated degradation of the mature form of SREBP1 in EGFRm NSCLC cells (16). Therefore, we suggest that osimertinib decreases Topo IIα levels via promoting GSK3-dependent and FBXW7-mediated proteasomal degradation in coordination with suppression of *SMURF2* expression in EGFRm NSCLC cell lines. Osimertinib is known to inhibit Akt phosphorylation or activity (16, 17). Since GSK3 is a well-known substrate of Akt, it is likely that osimertinib inhibits Akt-dependent GSK3 phosphorylation in EGFRm NSCLC cells, leading to GSK3 activation and subsequent GSK3-dependent and FBXW7-mediated degradation of Topo IIα. This speculation may need further investigation in the future.

In summary, our work has revealed an essential role of Topo IIα modulation in regulating the responses of EGFRm NSCLC cells to osimertinib; this provides a strong scientific rationale for managing acquired resistance to osimertinib and possibly other third-generation EGFR-TKIs via targeting Topo II. Our findings therefore warrant clinical validation of cotargeting EGFR and Topo II as an effective strategy to enhance the therapeutic efficacy of EGFR-targeted therapy and manage acquired resistance to osimertinib and other EGFR-TKIs.

## Methods

### Sex as a biological variable.

Lung cancer is not a sex-specific cancer. Human lung cancer tissues were collected from female and male patients. Both female and male mice were used in the study.

### Reagents.

Chemotherapeutic drugs, VP-16, DXR, PTX, CDDP, carboplatin, gemcitabine, 5-FU, cyclophosphamide, capecitabine, and vincristine were purchased from MedChemExpress. Topo IIα (catalog 12286), Smurf2 (catalog 12024), cPARP (catalog 5625), and Bim (catalog 2933) antibodies were purchased from Cell Signaling Technology Inc. Mcl-1 (sc-12756) and Topo IIβ (sc-55330) antibodies were purchased from Santa Cruz Biotechnology. Anti–phospho-histone H2AX (Ser139; γ-H2AX) antibody was purchased from MilliporeSigma (catalog 05–636). DAPI (catalog 62248) and secondary antibody Alexa Fluor 488 donkey anti-mouse (A32766) were purchased from Thermo Fisher Scientific. Other reagents and antibodies were the same as described previously (10, 16).

### Cell lines and cell culture.

All cell lines used in this study have been described previously (17, 29, 30). PC-9/AR/Bim-KO and HCC827/AR/Bim-KO cells were established using the same method as described in our previous study (29). Cell lines that stably overexpress the *TOP2A* gene were established with infection of lentiviruses carrying a human *TOP2A* gene that encodes Topo IIα protein followed by kanamycin selection. *TOP2A* lentiviral plasmid (catalog 444860610195) and matching vector pLenti-GIII-CMV were purchased from Applied Biological Materials Inc. and used as instructed by the manufacturer. These cell lines have not been genetically authenticated. All cell lines were cultured in RPMI 1640 medium supplemented with 5% FBS at 37°C in 5% CO_2_ humidified air.

### Colony formation assay.

The tested cell lines were seeded in 12-well plates at a density of 150 or 200 cells/well for 24 hours and then exposed to the tested drugs. The medium was replaced with fresh medium containing the same drugs every 3 days. After incubation for 10 days, the medium was removed. The plates were then fixed and stained with 2% crystal violet in ethanol for colony counting (> 50 cells/well) and photographing.

### Cell survival assay.

Cells were seeded in 96-well plates at appropriate densities. On the second day they were exposed to drug treatments either alone or in combination for 3 days. Cell numbers were measured by sulforhodamine B (SRB) assay as previously described (31). CI for drug interaction was calculated with the CompuSyn software (ComboSyn Inc.).

### Apoptosis assays.

Apoptosis was evaluated with the annexin V/7-AAD apoptosis detection kit (BD Biosciences) according to the manufacturer’s protocol. Apoptosis was also demonstrated by protein cleavages detected with Western blotting.

### Western blot analysis.

The procedures used for the preparation of the whole-cell protein lysates and immunoblotting were described previously (10). Protein band intensities were quantified by ImageJ (NIH) software.

### γ-H2AX foci assay.

Cells were seeded in chamber slides and then treated with the tested drugs. After different times, the cells were fixed with 4% paraformaldehyde for 10 minutes and then blocked with 5% BSA and 0.2% Triton X-100 in PBS buffer for 1 hour at room temperature. The cells were incubated with γ-H2AX antibody (1:100 dilution) at 4°C overnight and then incubated with the secondary antibody Alexa Fluor 488 donkey anti-mouse (1:200 dilution) for 2 hours at room temperature followed by DAPI (1:1,000) counterstaining. Images were collected using a confocal microscope (Leica TCS SP8).

### Protein stability assay.

Cells were treated with DMSO or osimertinib for a given time followed by addition of fresh medium with 10 μg/mL CHX and then harvested at different times for preparation of whole-cell protein lysates and subsequent Western blot analysis.

### Detection of DTCs.

Cells seeded in 12-well plates at a density of close to 90% were exposed to the tested drugs. The medium was replaced with fresh medium containing the same drugs every 2 days. After incubation for 5 or 10 days, the medium was removed for fixing and staining DTCs with 2% crystal violet in ethanol.

### Quantitative reverse transcription PCR.

Cellular total RNA was extracted using an RNA extraction kit (Qiagen) according to the manufacturer’s instructions. The concentrations were measured with NanoDrop (Thermo Fisher Scientific), and reverse transcription was carried out using the RevertAid First Strand cDNA Synthesis Kit (Qiagen). qPCRs were performed at 95°C for 15 seconds followed by 40 cycles of 95°C for 5 seconds and 60°C for 30 seconds using QuantStudio 3 and 5 systems (Thermo Fisher Scientific). GAPDH was used as an endogenous control. The following primers were used: TOP2A, 5′-GTGGCAAGGATTCTGCTAGTCC-3′ (forward) and 5′-ACCATTCAGGCTCAACACGCTG-3′(reverse); GAPDH, 5′-GTCTCCTCTGACTTCAACAGCG-3′ (forward) and 5′-ACCACCCTGTTGCTGTAGCCAA-3′ (reverse).

### Gene knockdown using siRNA and shRNA.

*TOP2A* siRNA (sc-36695), *TOP2A* shRNA plasmid (sc-36695-SH), *SMURF2* siRNA (sc-41675), and *SMURF2* shRNA plasmid (sc-41675-SH) were purchased from Santa Cruz Biotechnology. Scrambled control, GSK3α/β and FBXW7 siRNAs, FBXW7 shRNA, and the procedures used for transfection were described previously (16).

### TCGA data analysis.

The comparison of *TOP2A* expression between lung adenocarcinoma (*n* = 483) and normal (*n* = 347) samples was conducted using GEPIA2 (32), where lung adenocarcinoma samples were from TCGA and normal samples were from both TCGA and Genotype-Tissue Expression (GTEx; https://gtexportal.org/home/) data. Kaplan-Meier analysis was performed using data from patients treated with EGFR TKIs retrieved from TCGA lung adenocarcinoma data sets (http://cancergenome.nih.gov/). Patients were stratified according to high versus low expression (cutoff: median) of *TOP2A* within their tumors.

### Human NSCLC tissues.

Paired tissue samples from patients with EGFRm NSCLC before treatment (i.e., baseline) and after disease relapse from treatment with first-generation EGFR-TKIs, including gefitinib, erlotinib, icotinib, or osimertinib, were collected at the Second Xiangya Hospital and Henan Cancer Hospital under ethics review committee–approved protocols. All tissues were sent to and stained at the Second Xiangya Hospital.

### IHC.

Topo IIα in human NSCLC tissues was stained with IHC using the EnVision + Dual Link System-HRP Kit (Dako). After tissue sections were deparaffinized and rehydrated, high-temperature antigen retrieval was achieved by heating the samples in EDTA (1:50, pH 9.0) with a pressure cooker for 7 minutes followed by an incubation with 3% H_2_O_2_ to block endogenous peroxidase activity for 30 minutes. The tissue sections were then incubated with the ready-to-use Topo IIα antibody (catalog MAB-7099; Maixin Biotech) overnight at 4°C followed by an incubation with secondary antibody (MaxVision TM HRP-Polymer anti-Mouse/Rabbit IHC Kit, catalog KIT-5030; Maixin Biotech) at room temperature for 30 minutes. Color reaction was developed by using 3,3′-diaminobenzidine tetrachloride chromogen solution. All slides were counterstained with hematoxylin. Positive control slides were included in every experiment in addition to the internal positive controls. The percentage of positive staining in tumor cells was scored. The staining of slides with xenograft tumor tissues was the same as described previously (16). The dilutions of antibodies used were 1:100 (Topo IIα), 1:50 (cPARP), 1: 200 (Bim), and 1:100 (Mcl-1), respectively.

### Animal xenograft and treatments.

In the conventional cell–derived xenograft studies, cells suspended in sterile PBS at 3 × 10^6^ cells per mouse were injected into the flanks of 4-week-old nu/nu nude mice purchased from The Jackson Laboratory. On day 7, when the average tumor was around 80 mm^3^, the mice were divided into groups with equal average tumor volumes and body weights. The following treatments were administered daily: vehicle, osimertinib (5 mg/kg, oral gavage), VP-16 (1 mg/kg, i.p.), DXR (2 mg/kg, i.p.), and the combination of osimertinib and VP-16 or DXR. Tumor volume was measured using calipers every 2 or 3 days and calculated as *V* = π(length × width^2^)/6. Body weight was also measured every 2 or 3 days. At the end of the experiment, mice were sacrificed using CO_2_. The tumors were then removed, weighed, and stored in formalin for further analysis. The same treatments were also applied to the immunocompetent C57BL/6J mice, which were purchased from The Jackson Laboratory. Evaluation of major mouse organ tissues with H&E staining was conducted in Cancer Tissue and Pathology Shared Resource of Winship Cancer Institute at Emory University. Biochemical tests of serum protein markers and blood cell counting were performed in the Department of Pathology at the College of Veterinary Medicine, University of Georgia (Athens, Georgia, USA).

In PDX studies, the 3 PDXs harboring different EGFR mutations, TM00193 (E746_A750del), TM00199 (L858R), and TM00219 (E746_A750del; T790M; exon 19 del), were purchased from The Jackson Laboratory. When the average tumor was around 100 mm^3^, the mice were treated with vehicle, osimertinib (5 mg/kg, oral gavage), VP-16 (1 mg/kg, i.p.), and the combination of osimertinib with VP-16 every day. Tumor volume was measured using calipers every 3 or 4 days. At the end of the experiment, mice were sacrificed using CO_2_. The tumors were then removed, weighed, and stored in formalin for further analysis.

### Statistics.

Statistical differences between 2 groups were determined by 2-tailed unpaired or paired Student’s *t* test. One-way ANOVA was conducted to evaluate differences among multiple groups. Results are presented as mean ± SD or SEM. All statistical analyses were conducted using Graphpad Prism 9.0 software. *P* values of less than 0.05 were considered statistically significant.

### Study approval.

Animal experiments were approved by the Institutional Animal Care and Use Committee of Emory University. Human lung cancer tissue collection was conducted under ethics review committee–approved (IRB-approved) protocols (The Second Xiangya Hospital, IRB2019-009; Henan Cancer Hospital, IRB2019-067; and Emory University School of Medicine, IRB00104138).

### Data availability.

All data for in this study are included in the article or supplemental material and are available upon reasonable request; values for all data points in graphs are reported in the Supporting Data Values file.

## Author contributions

ZC conceptualized the study; provided methodology, investigation, formal analysis, data curation, and visualization; and wrote the original draft of the manuscript. KAV provided investigation and resources and wrote, reviewed, and edited the manuscript. DW provided investigation. ZQ provided investigation and formal analysis. SF provided formal analysis, resources, and supervision and wrote, reviewed, and edited the manuscript. QW provided resources and wrote, reviewed, and edited the manuscript. TL acquired funding and wrote, reviewed, and edited the manuscript. SSR acquired funding and wrote, reviewed, and edited the manuscript. SYS conceptualized the study; provided formal analysis, visualization, supervision, funding acquisition, and project administration; and wrote the original draft of the manuscript.

## Supplementary Material

Supplemental data

Unedited blot and gel images

Supporting data values

## Version 1 03/07/2024

In-Press Preview

## Version 2 05/15/2024

Electronic publication
