# Supplementary material for: DNA topoisomerase II inhibition potentiates osimertinib’s therapeutic efficacy in EGFR-mutant non–small cell lung cancer models
Source: J Clin Invest. 2024 Mar 7;134(10):e172716. doi: 10.1172/JCI172716 (PMC11093598; doi:10.1172/JCI172716)
Supplement: Supplemental data [file jci-134-172716-s105.pdf]

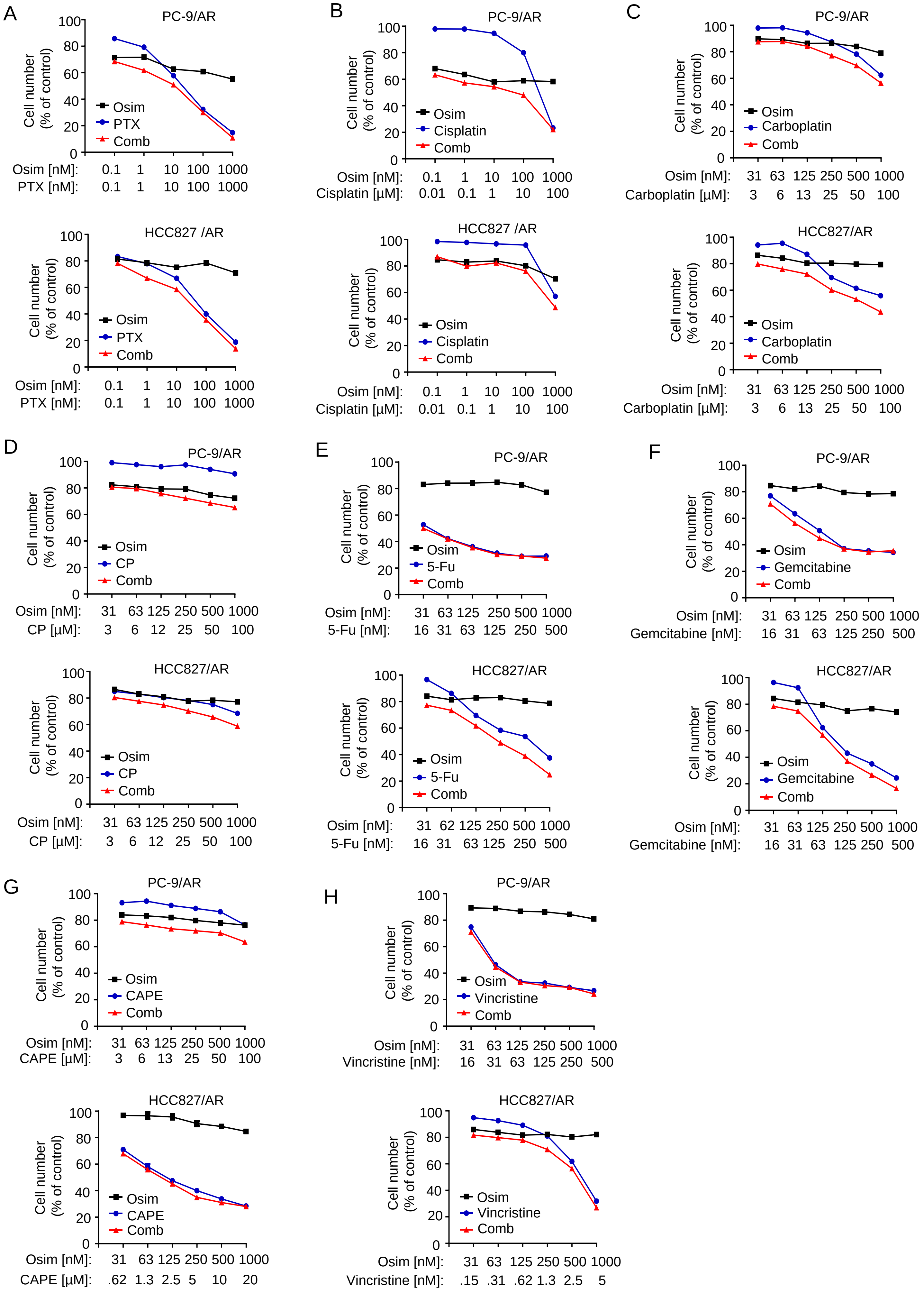

Fig. S1. Effects of osimertinib combined with different chemotherapeutic agents on the growth of osimertinib-resistant cells. Both PC-9/AR and HCC827 cells were exposed to the indicated treatments either as single agents or in combination for 3 days. Cell numbers were then measured with the SRB assay. The data are means ± SDs of four replicate determinations. PTX, paclitaxel; CP, cyclophosphamide; CAPE, capecitabine.

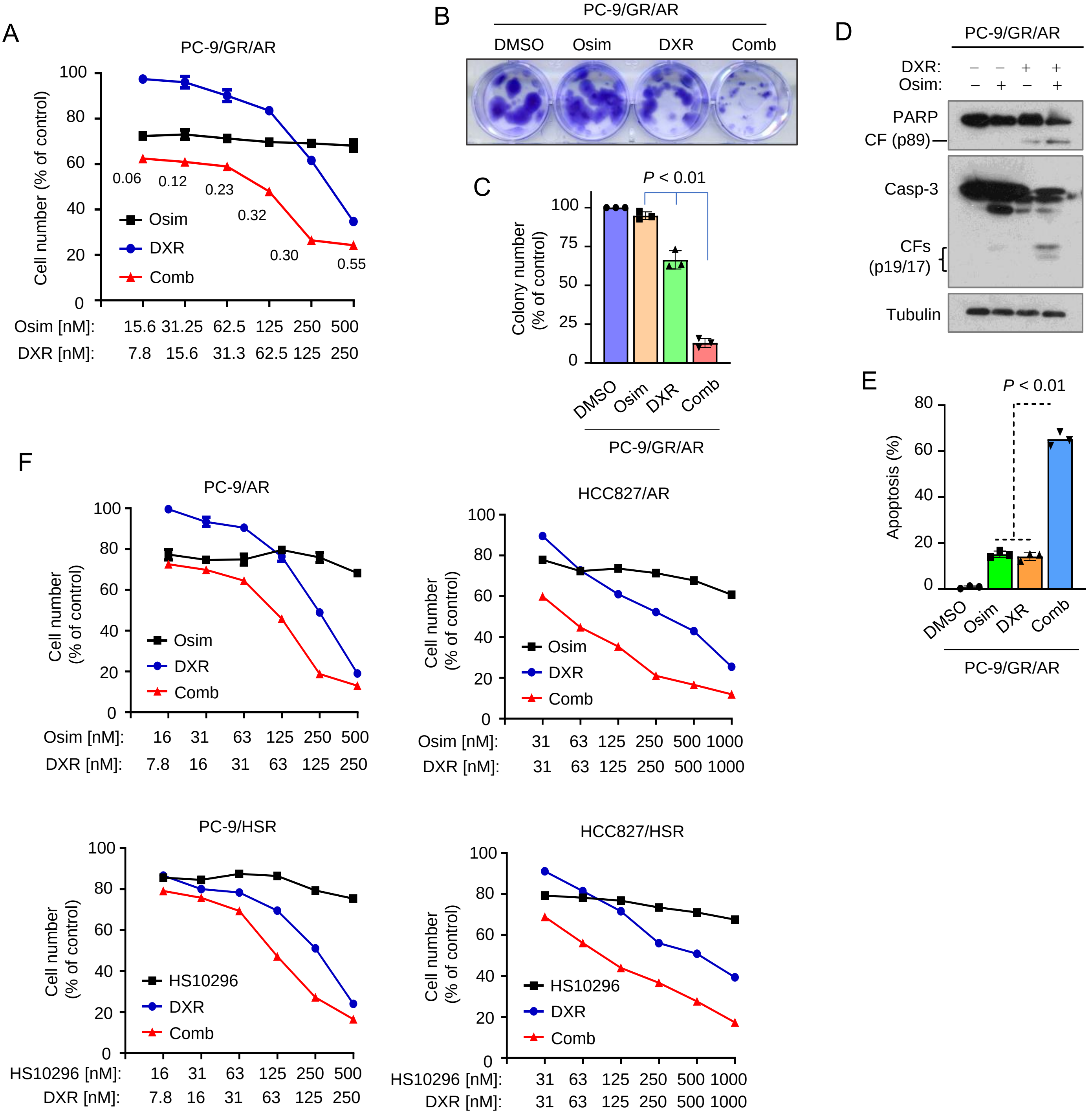

Fig. S2. Osimertinib or HS-10296 combined with DXR synergistically decreases cell survival with enhanced induction of apoptosis in EGFRm NSCLC with acquired resistance to osimertinib or HS-10296. *A* and *F*, The given cell lines were exposed to the indicated agents either alone or in combination for 3 days. Cell numbers were determined with the SRB assay. *B* and *C*, PC-9/GR/AR cells in 12-well plates were treated with 50 nM Osim, 20 nM DXR or their combination every 3 days with fresh medium for a total of 10 days. Cell colonies were stained with crystal violet, photographed (*B*) and counted (*C*). *D* and *E*, PC-9/GR/AR cells were treated with 250 nM Osim, 100 nM DXR or their combination for 48 h and then harvested for detection of protein cleavage (*D*) with Western blotting and annexin V-positive cells (*E*) with flow cytometry. The data are means ± SDs of four replicate (*A* and *F*) or triplicate (C and *E*) determinations. Statistical differences among multiple groups were evaluated with one-way ANOVA test.

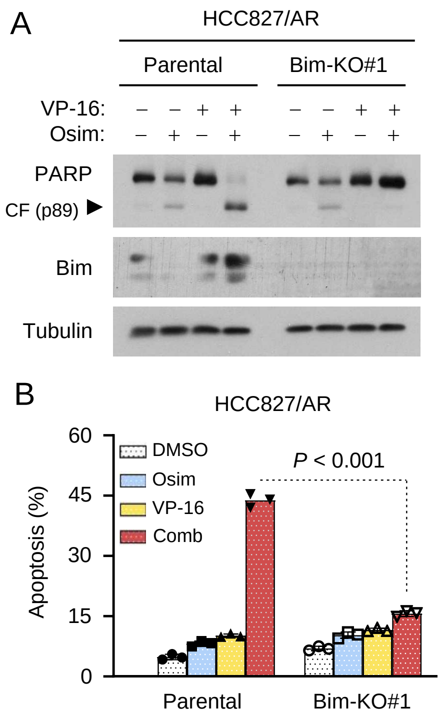

Fig. S3. Bim knockout in HCC827/AR cells compromises the effects of Osim on induction of apoptosis. The given cell lines were exposed to DMSO, 250 nM Osim, 1 μM VP-16 for 48 h. The proteins of interest were detected with Western blotting (*A*) and annexin V-positive cells were measured with flow cytometry (*B*). Each column represents the mean ± SD of triplicate determinations. CF, cleaved form. Statistical difference between two groups was conducted with two-sided unpaired Student’s t-test.

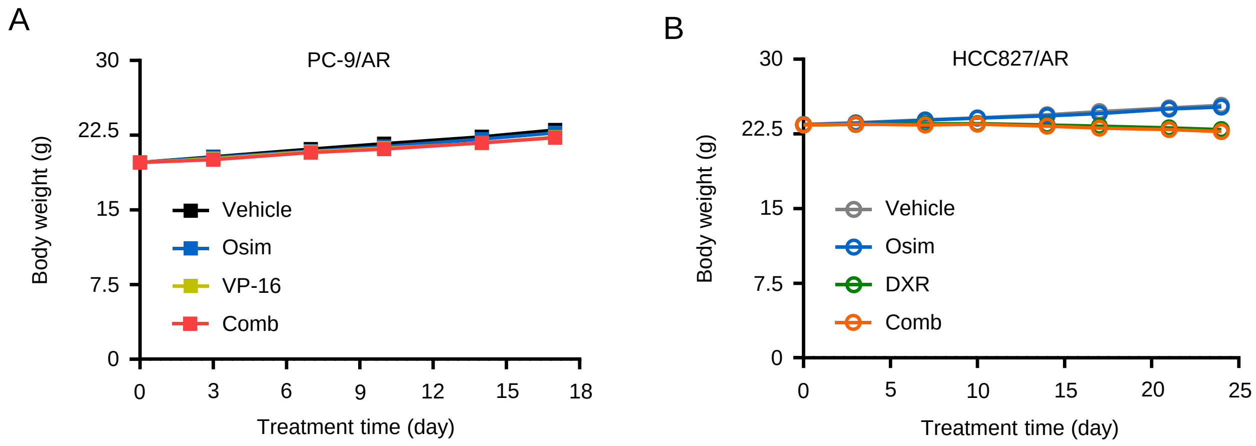

Fig. S4. The combination of osimertinib (Osim) with VP-16 (A) or DXR (B) is well tolerated in nude mice. Treatments were the same as described in Fig. 2.

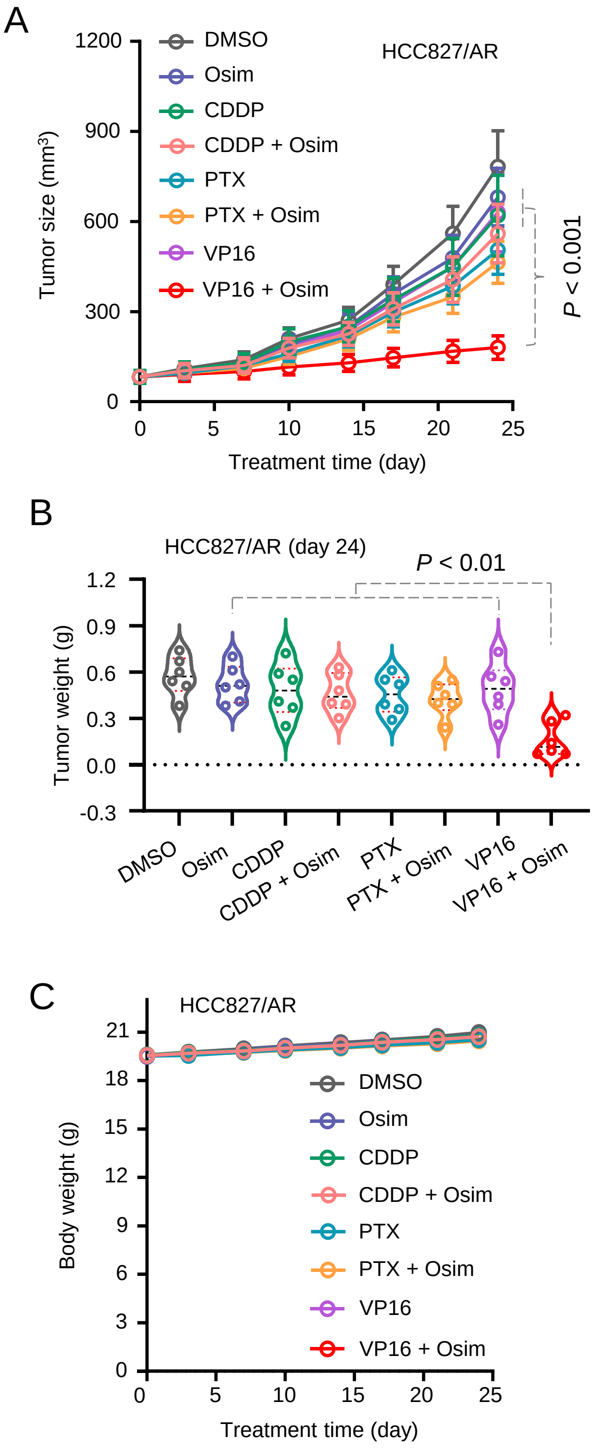

Fig. S5. VP-16, but not CDDP or PTX, when combined with osimertinib (Osim), significantly inhibits the growth of osimertinib-resistant tumors *in vivo*. HCC27/AR cells grown in NU/NU mice as xenografted tumors (n = 6/group) were treated with vehicle, osimertinib alone (5 mg/kg, daily, og), CDDP alone (3 mg/kg/day, daily, ip), PTX alone (1 mg/kg/day, daily, ip), VP-16 alone (1 mg/kg/day, daily, ip) or the indicated combinations. Tumor sizes were measured at the indicated time points (*A*). At the end of treatment, tumors (*B*) and body weight (*C*) in each group were also weighed. The data in each group are means ± SEs of 6 tumors from 6 mice. Statistical differences were conducted with one-way ANOVA test.

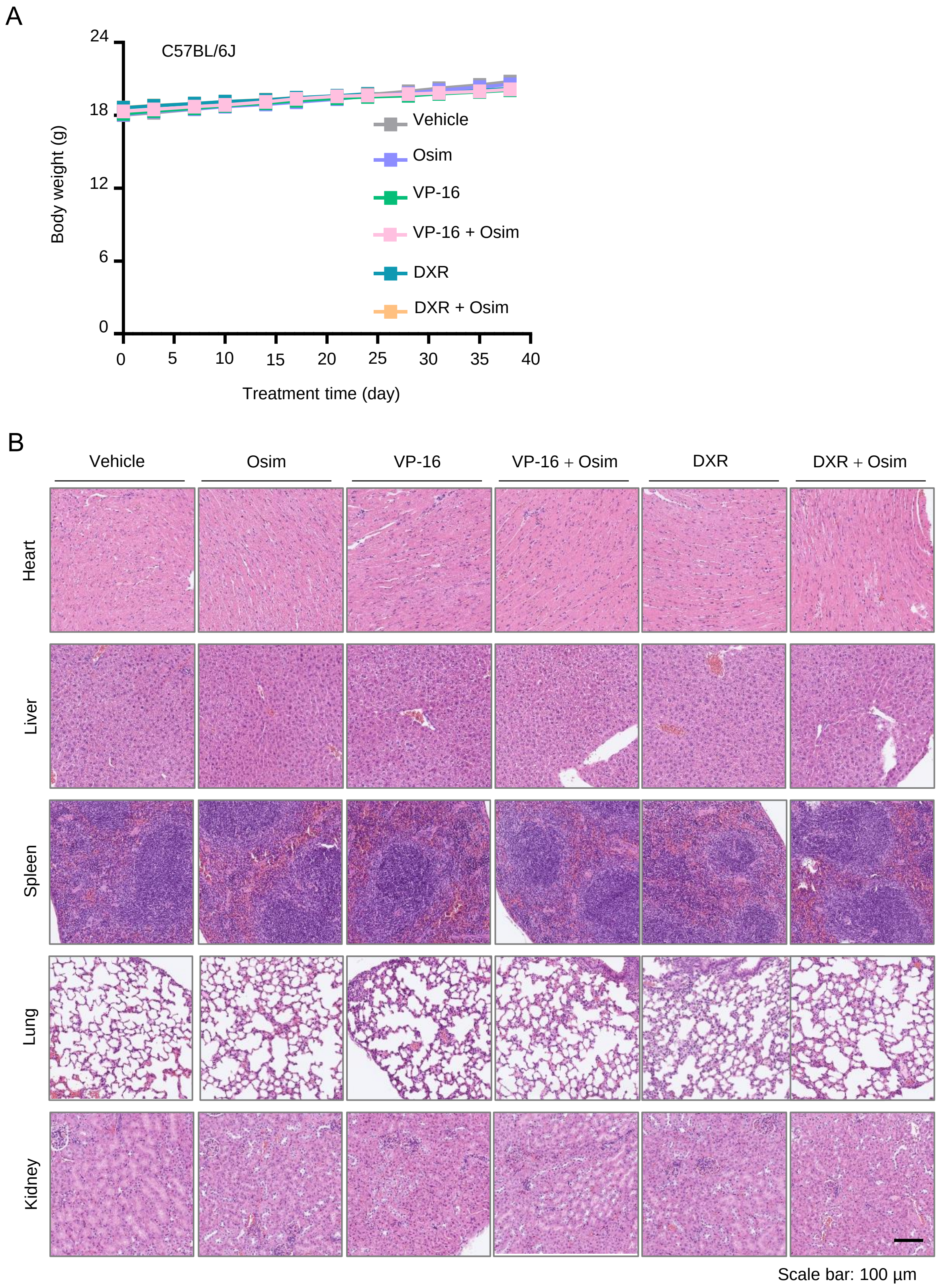

Fig. S6A and B. The combination of osimertinib (Osim) with VP-16 or DXR is well tolerated in immunocompetent mice in terms of body weight alterations (*A*) and histological examination of key organ tissues (*B*). Treatments were the same as described in Fig. 2.

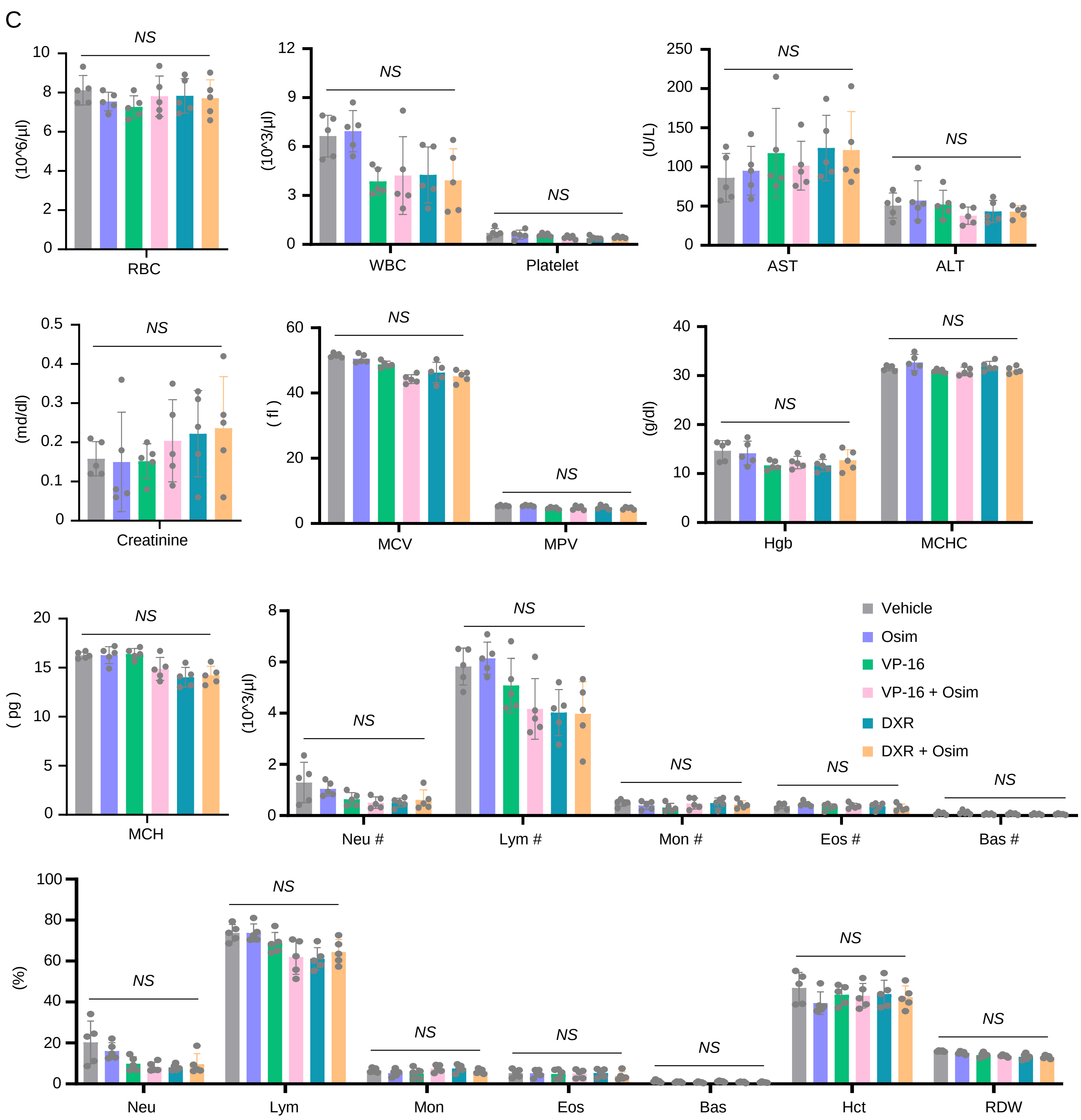

Fig. S6C. The combination of osimertinib (Osim) with VP-16 or DXR is well tolerated in immunocompetent mice in terms of evaluating blood cells and serum markers. Treatments were the same as described in Fig. 2. NS, not significant evaluated with one-way ANOVA test; RBC, red blood cell; WBC, white blood cells; AST, Aspartate aminotransferase; ALT, Alanine transaminase; MCV, mean corpuscular volume; MPV, mean platelet volume; Hgb, hemoglobin; MCHC, mean corpuscular hemoglobin concentration; MCH, mean corpuscular hemoglobin; Neu, neutrophils; Lym, lymphocytes; Mon, monocytes; Eos, eosinophil; Bas, basophil; Hct, hematocrit; RDW, red blood cell distribution width.

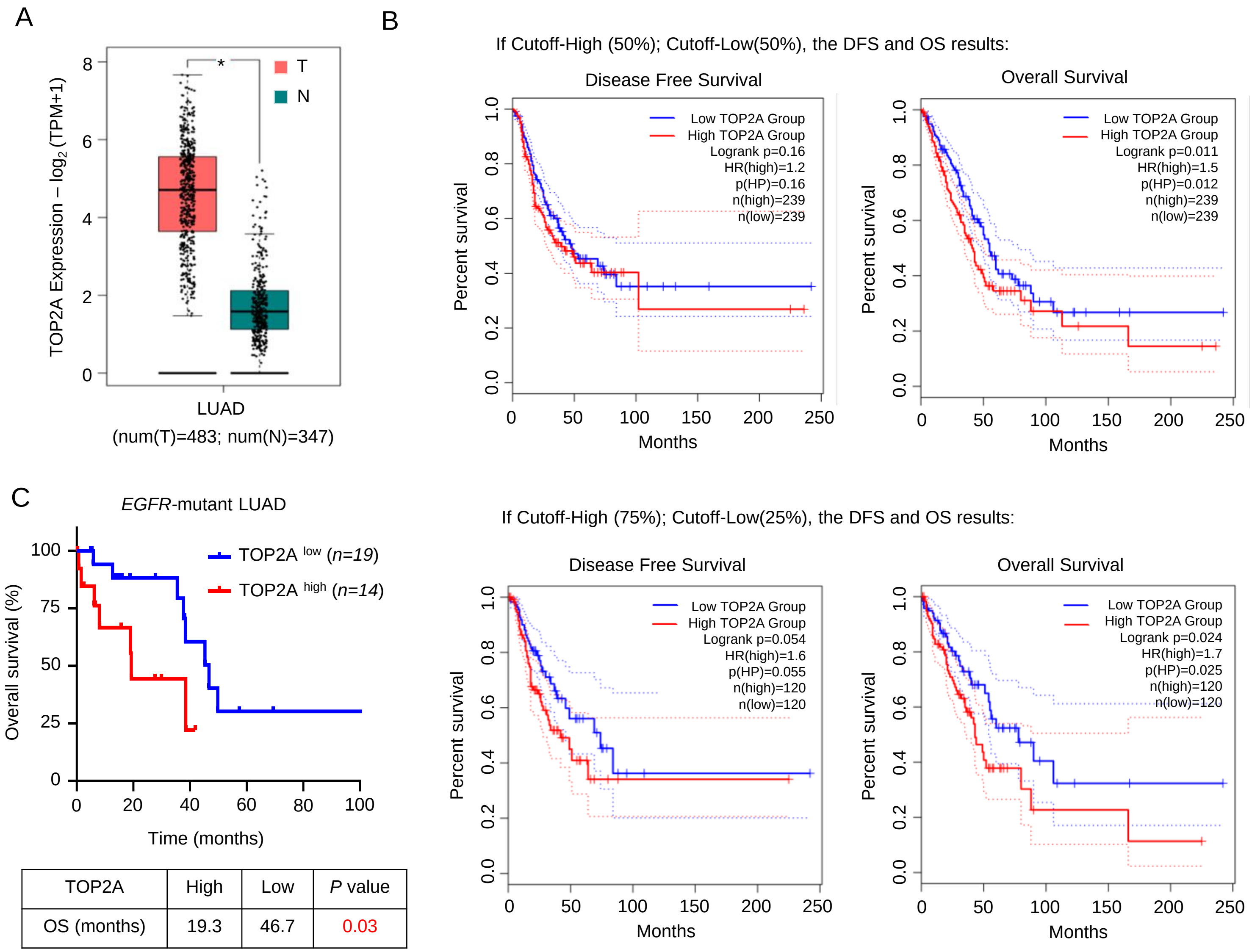

TOP2A	High	Low	*P* value
OS (months)	19.3	46.7	0.03

Fig. S7. TCGA data analysis of *TOP2A* expression (*A*) and its impact on cell survival (*B* and *C*) in lung adenocarcinomas (LUAD).

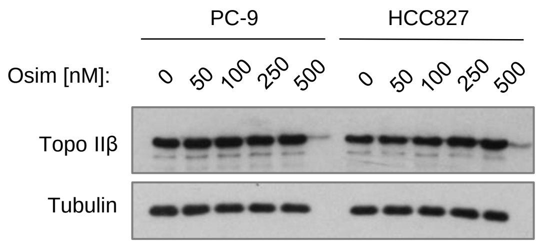

Fig. S8. Osimertinib does not decrease Topo IIβ levels in EGFRm NSCLC cells. The tested cell lines were exposed to the indicated concentrations of osimertinib (Osim) for 24 h. The proteins of interest in whole cell protein lysates were detected with Western blotting.

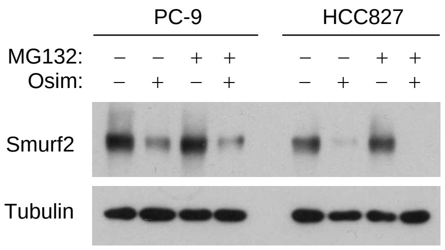

Fig. S9. Inhibition of proteasome does not rescue the reduction of Smurf2 protein by osimertinib in EGFRm NSCLC cells. Both PC-9 and HCC827 cells were pre-treated with 10 µM MG132 for 30 min and then co-treated with DMSO or 200 nM osimertinib (Osim) for another 6 h. The proteins of interest were detected with Western blotting.

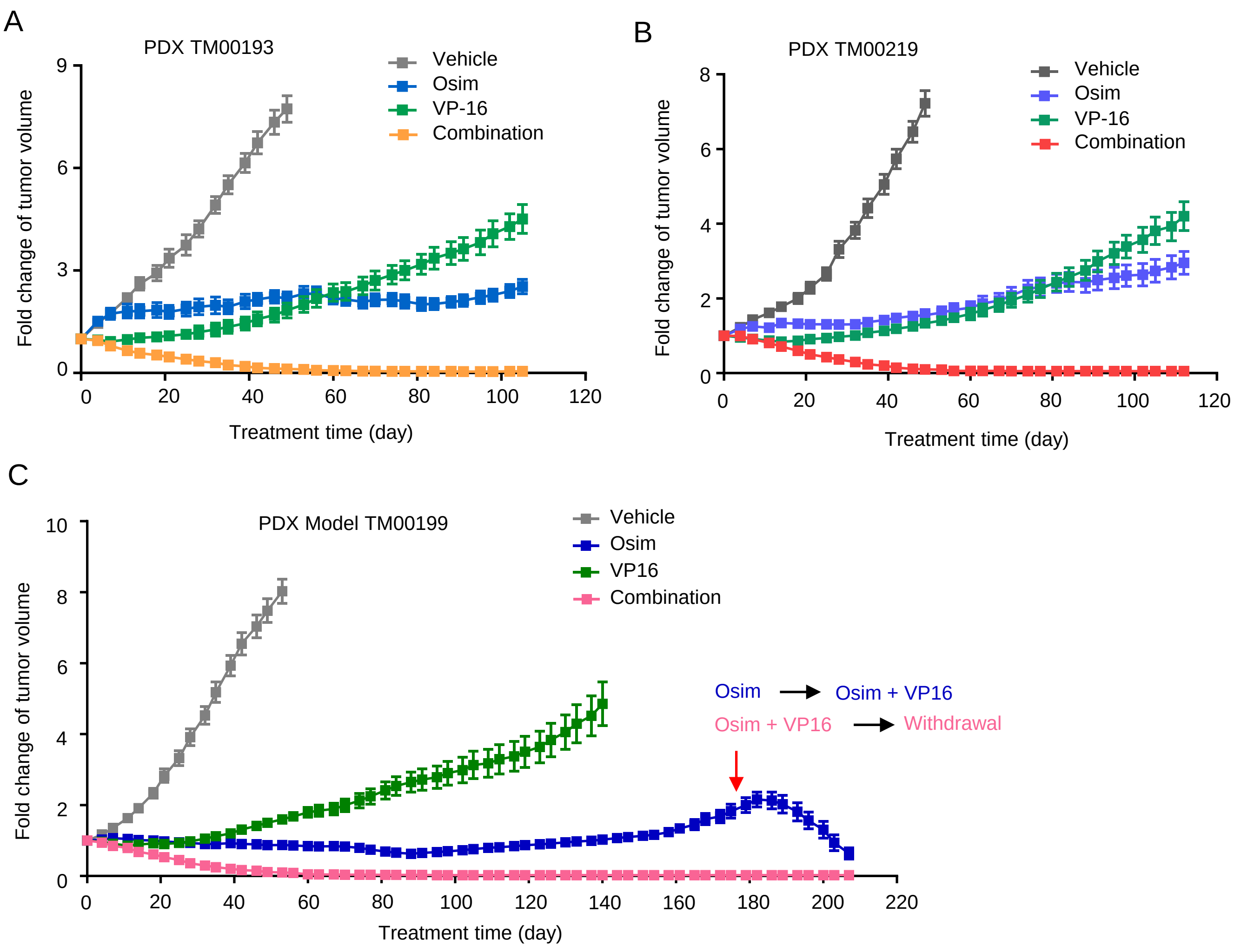

Fig. S10. The combination of osimertinib (Osim) and VP-16 potentiates effects on EGFRm NSCLC tumor regression and delays tumor relapse. Treatments were the same as described in Fig. 7. Tumor growth is presented as fold change in tumor sizes in comparison with their initial sizes of PDXs. Red arrow indicates treatment switches.

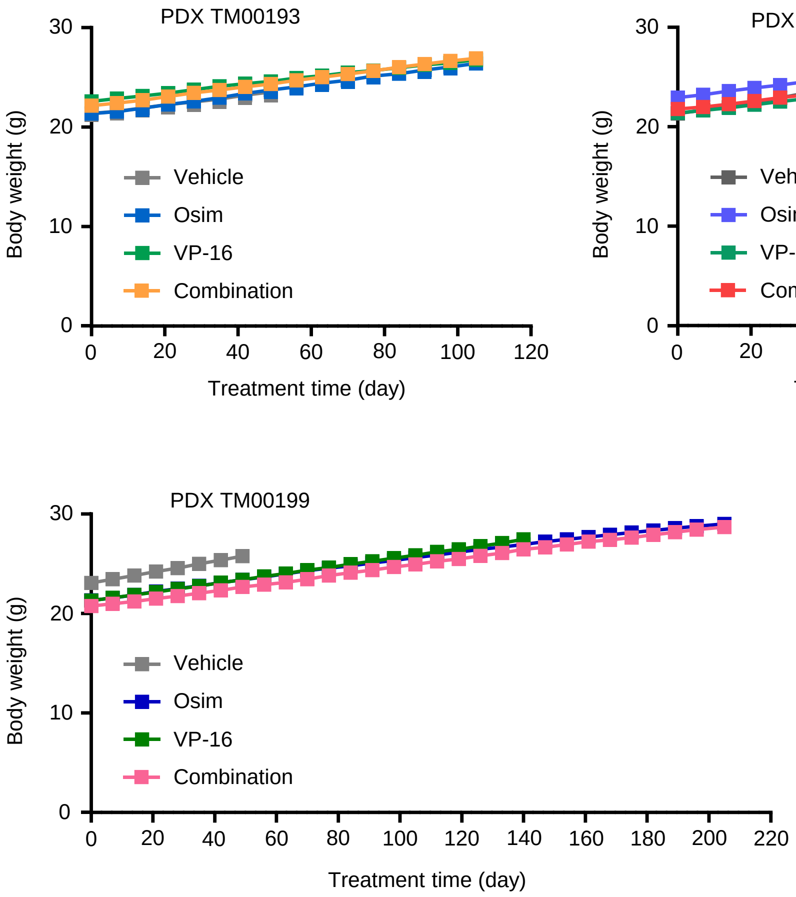

Fig. S11. The combination of osimertinib (Osim) and VP-16 is well tolerated in nude mice without enhanced toxicity. Treatments were the same as described in Fig. 7.
