# Supplementary material for: DNA topoisomerase II inhibition potentiates osimertinib’s therapeutic efficacy in EGFR-mutant non–small cell lung cancer models
Source: J Clin Invest. 2024 Mar 7;134(10):e172716. doi: 10.1172/JCI172716 (PMC11093598; doi:10.1172/JCI172716)
Supplement: Unedited blot and gel images [file jci-134-172716-s106.pdf]

PARP

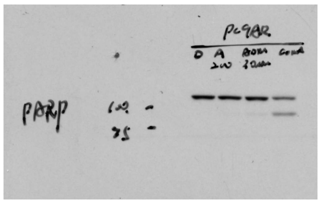

Full unedited gel for Figure 1F

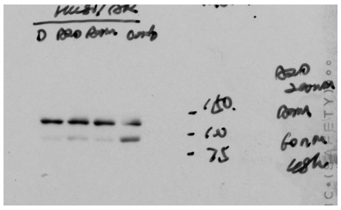

Full unedited gel for Figure 1F

PARP

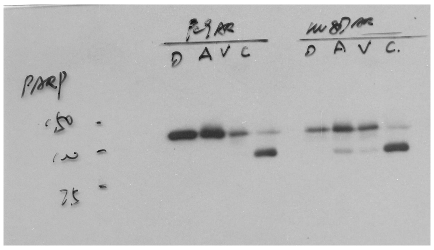

Full unedited gel for Figure 1F

Caspase 3

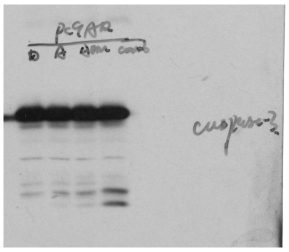

Full unedited gel for Figure 1F

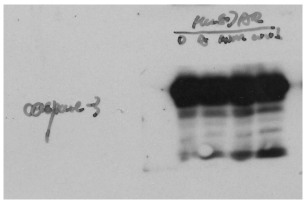

Full unedited gel for Figure 1F

Caspase 3

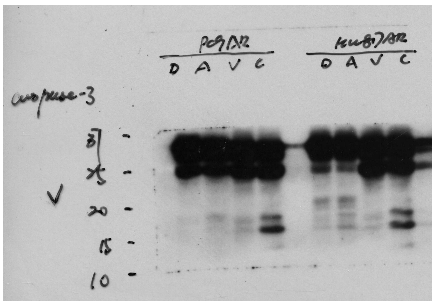

Full unedited gel for Figure 1F

Tubulin

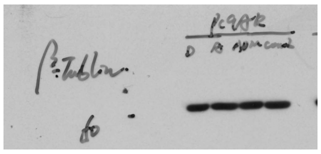

Full unedited gel for Figure 1F

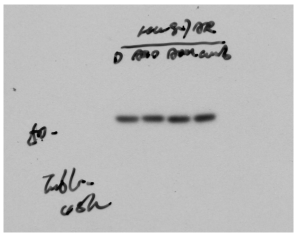

Full unedited gel for Figure 1F

Tubulin

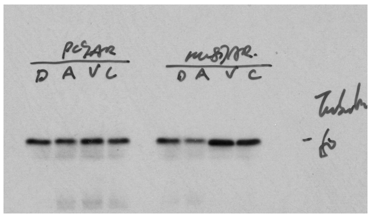

Full unedited gel for Figure 1F

Bim

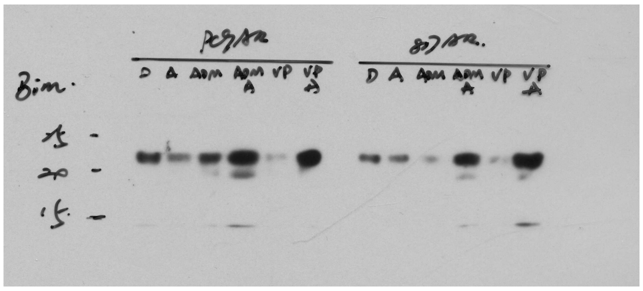

Full unedited gel for Figure 1H

PARP

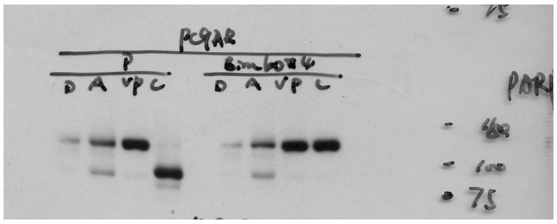

Full unedited gel for Figure 1I

Mci-1

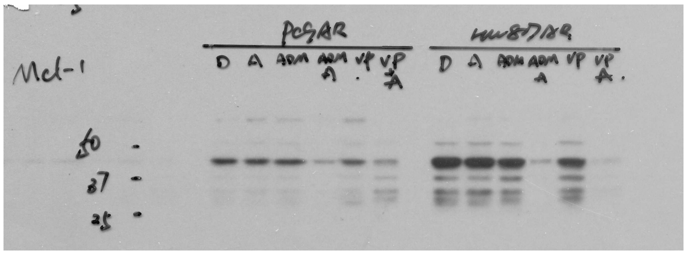

Full unedited gel for Figure 1H

Bim

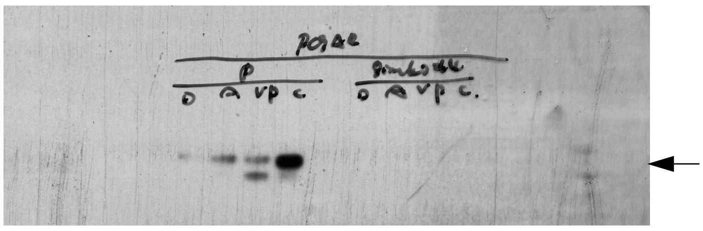

Full unedited gel for Figure 1I

Tubulin

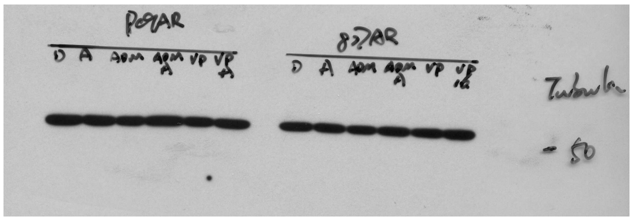

Full unedited gel for Figure 1H

Tubulin

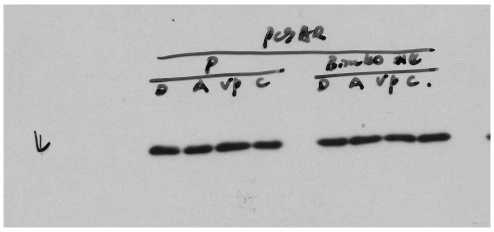

Full unedited gel for Figure 1I

Fig 1

TOP2α

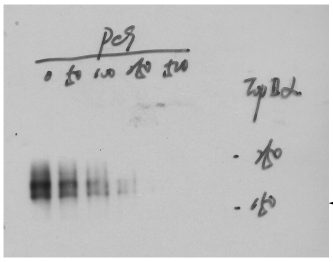

Full unedited gel for Figure 3A

TOP2α

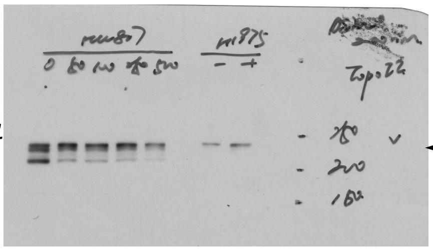

Full unedited gel for Figure 3A

Tubulin

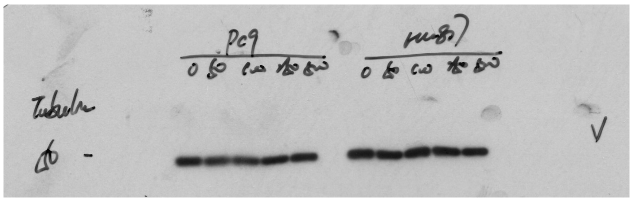

Full unedited gel for Figure 3A

TOP2α

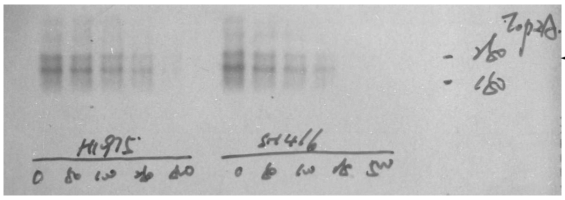

Full unedited gel for Figure 3A

Tubulin

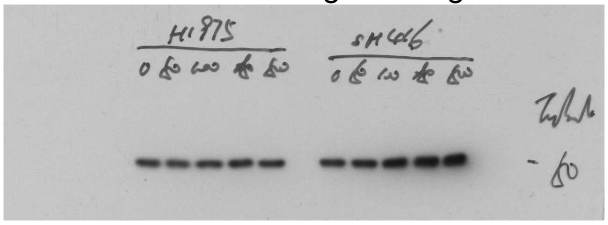

Full unedited gel for Figure 3A

TOP2α

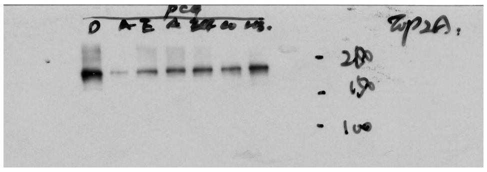

Full unedited gel for Figure 3C

TOP2α

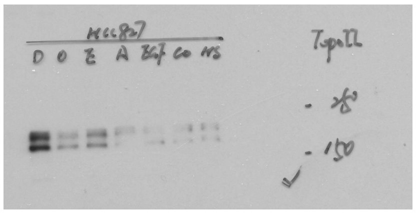

Full unedited gel for Figure 3C

Tubulin

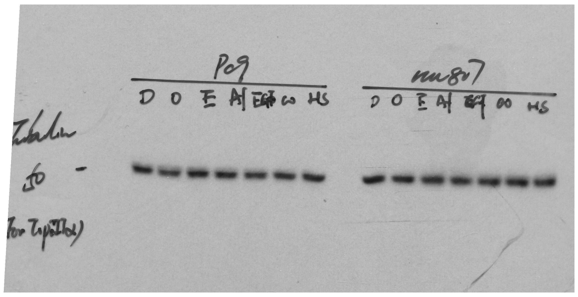

Full unedited gel for Figure 3C

TOP2α

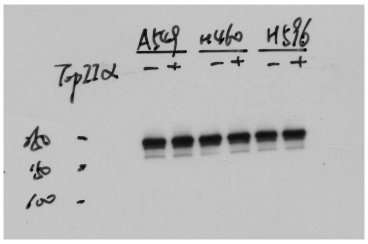

Full unedited gel for Figure 3D

Tubulin

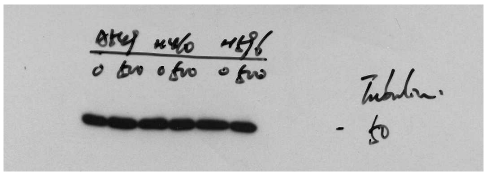

Full unedited gel for Figure 3D

TOP2α

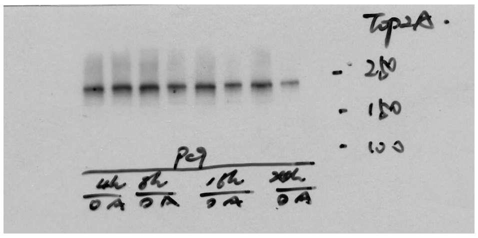

Full unedited gel for Figure 3B

Tubulin

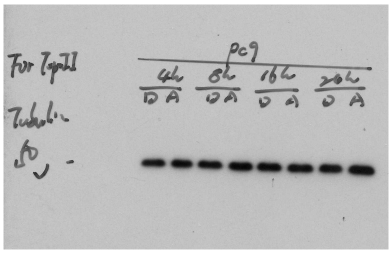

Full unedited gel for Figure 3B

TOP2α

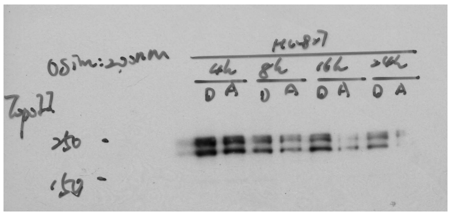

Full unedited gel for Figure 3B

Tubulin

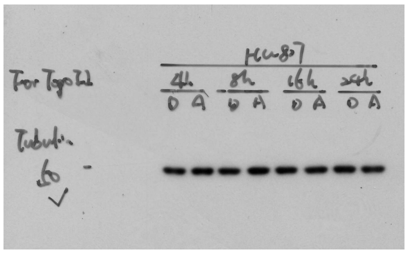

Full unedited gel for Figure 3B

PARP

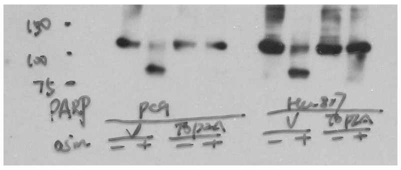

Full unedited gel for Figure 3G

TOP2α

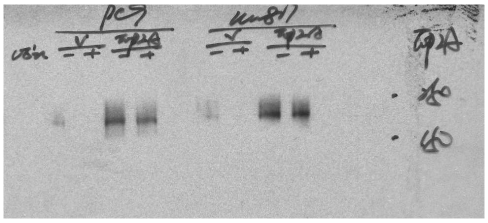

Full unedited gel for Figure 3G

Tubulin

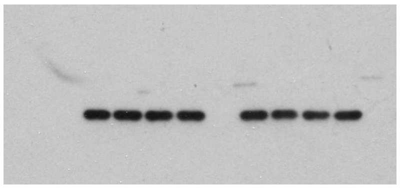

Full unedited gel for Figure 3G

Fig 3

OP2α

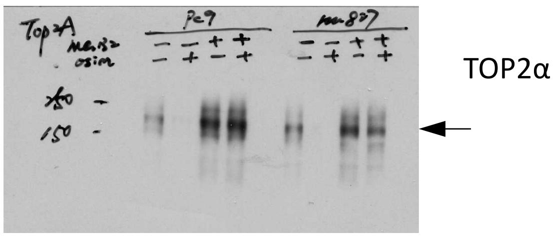

bulin

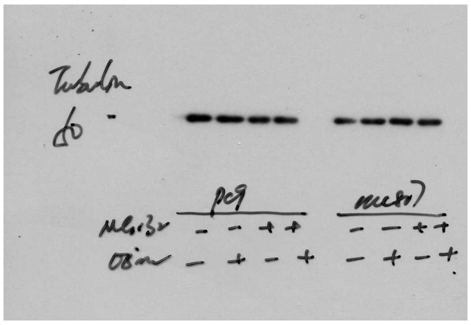

Full unedited gel for Figure 4B

Sumrf2

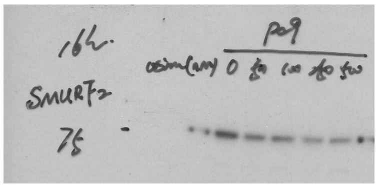

Full unedited gel for Figure 4E

Sumrf2

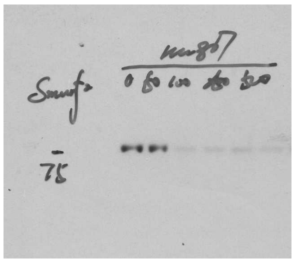

Full unedited gel for Figure 4E

GAPDH

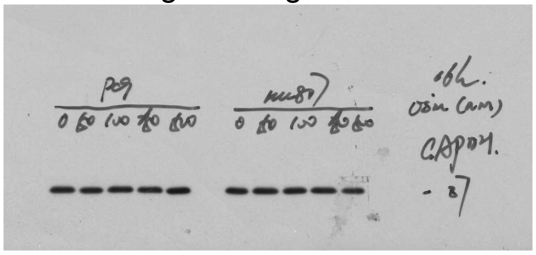

Full unedited gel for Figure 4E

TOP2α

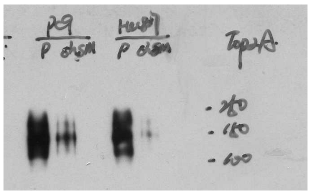

Full unedited gel for Figure 4G

Sumrf2

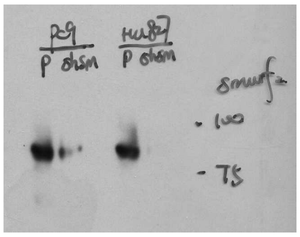

Full unedited gel for Figure 4G

Tubulin

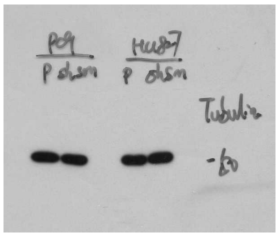

Full unedited gel for Figure 4G

TOP2α

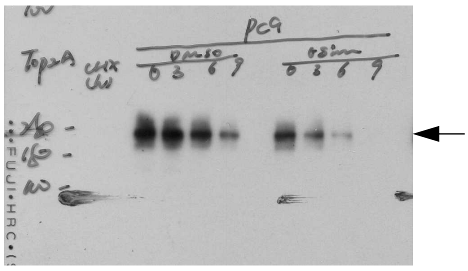

Full unedited gel for Figure 4C

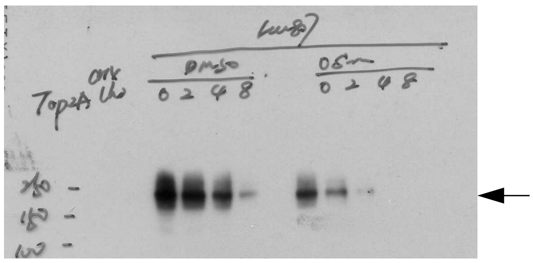

Full unedited gel for Figure 4C

Tubulin

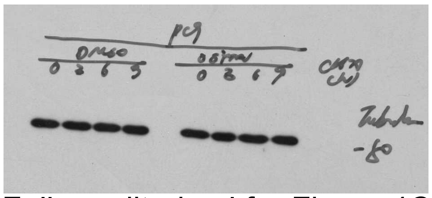

Full unedited gel for Figure 4C

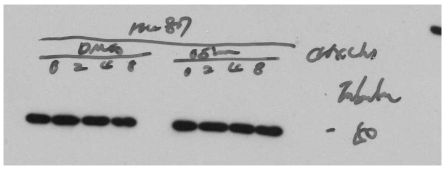

Full unedited gel for Figure 4C

TOP2α

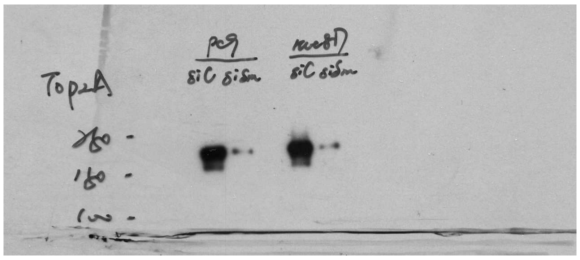

Full unedited gel for Figure 4F

Sumrf2

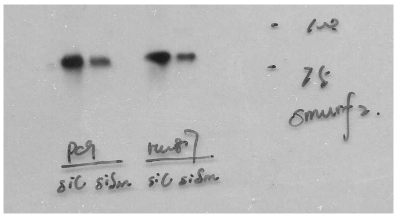

Full unedited gel for Figure 4F

Tubulin

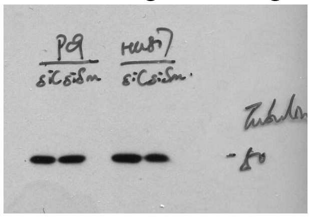

Full unedited gel for Figure 4F

TOP2α

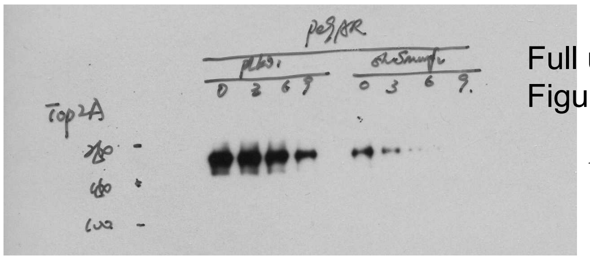

Full unedited gel for Figure 4H

Tubulin

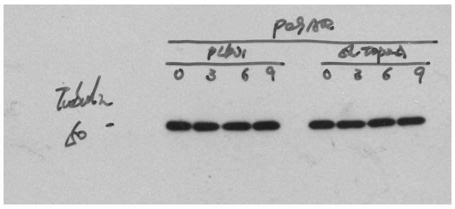

Full unedited gel for Figure 4H

TOP2α

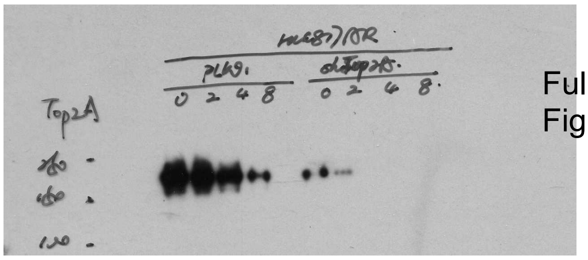

Full unedited gel for Figure 4H

Tubulin

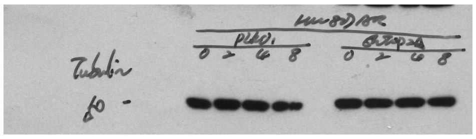

Full unedited gel for Figure 4H

Fig 4 (B-H)

TOP2α

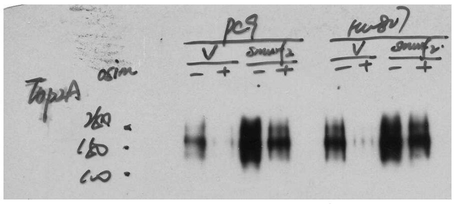

Full unedited gel for Figure 4I

TOP2α

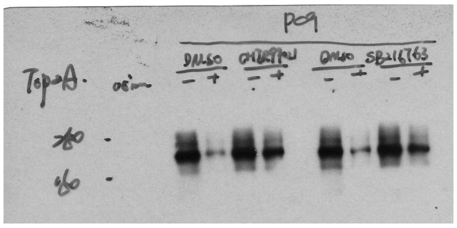

Full unedited gel for Figure 4J

Sumrf2

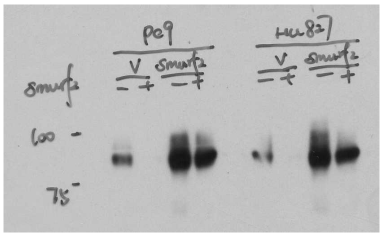

Full unedited gel for Figure 4I

Tubulin

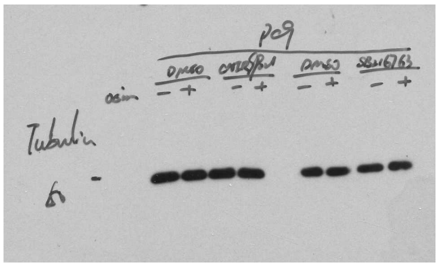

Full unedited gel for Figure 4J

Tubulin

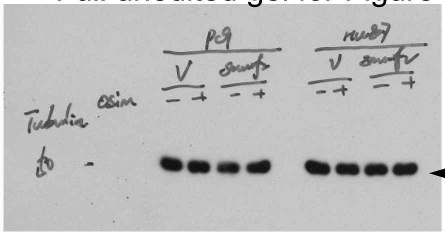

Full unedited gel for Figure 4I

TOP2α

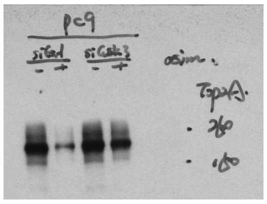

Full unedited gel for Figure 4K

TOP2α

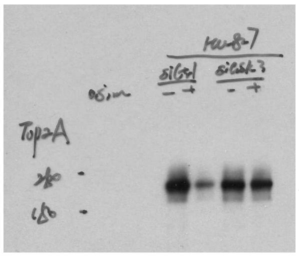

Full unedited gel for Figure 4K

TOP2α

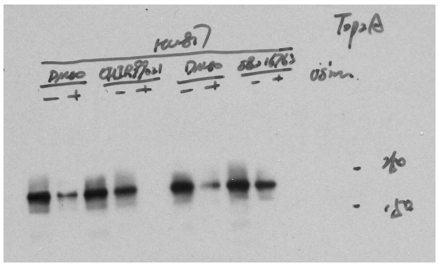

Full unedited gel for Figure 4J

GSK3α/β

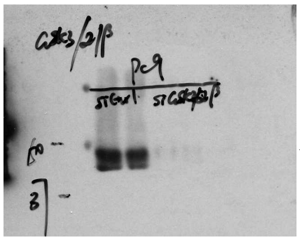

Full unedited gel for Figure 4K

GSK3α/β

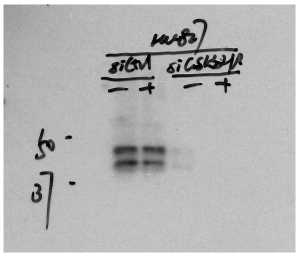

Full unedited gel for Figure 4K

Tubulin

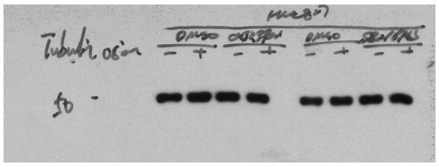

Full unedited gel for Figure 4J

Tubulin

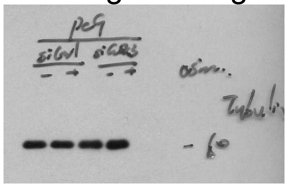

Full unedited gel for Figure 4K

Tubulin

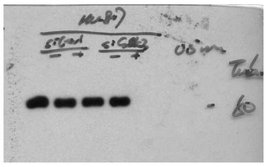

Full unedited gel for Figure 4K

TOP2α

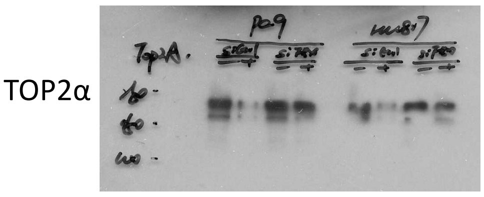

Full unedited gel for Figure 4M

TOP2α

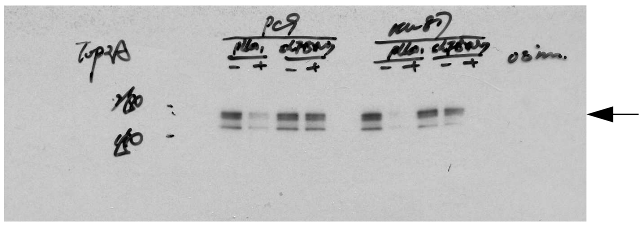

Full unedited gel for Figure 4N

FBXW7

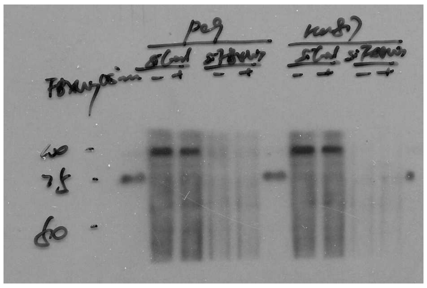

Full unedited gel for Figure 4M

FBXW7

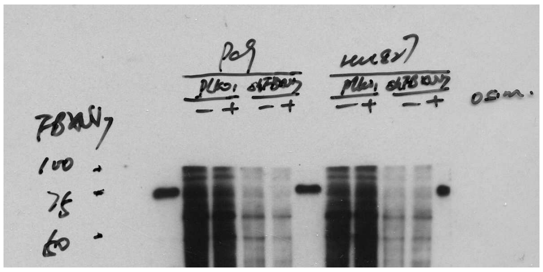

Full unedited gel for Figure 4N

Tubulin

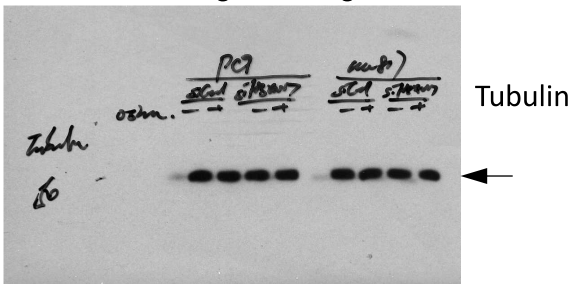

Full unedited gel for Figure 4M

Tubulin

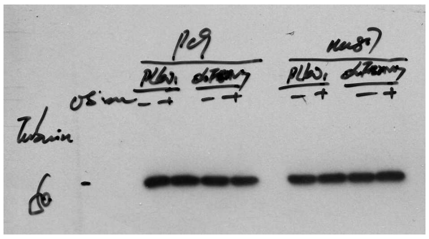

Full unedited gel for Figure 4N

Fig 4 (I-N)

TOP2α

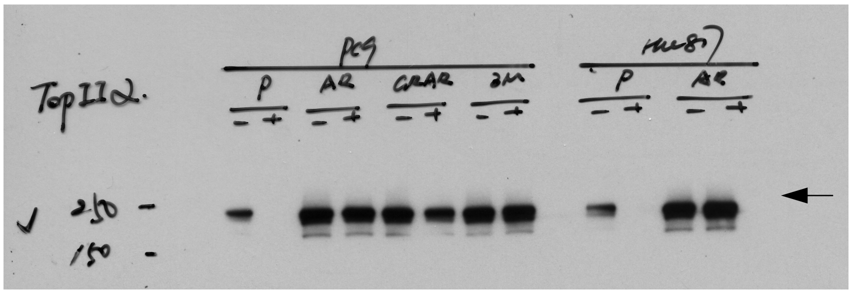

Full unedited gel for Figure 5A

TOP2α

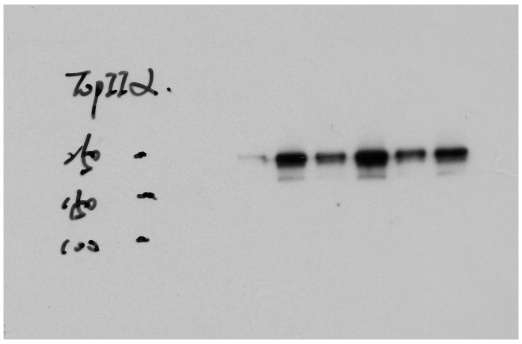

Full unedited gel for Figure 5B

Tubulin

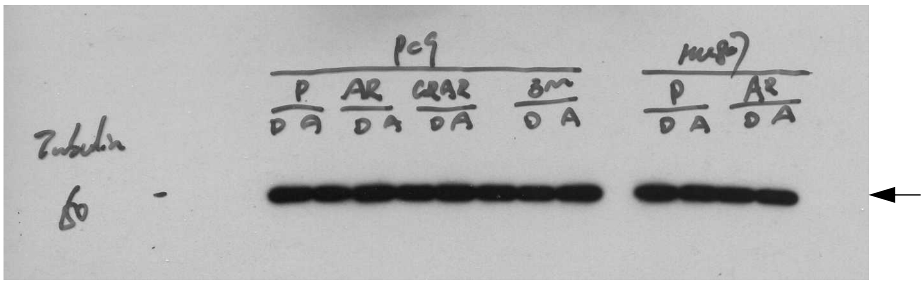

Full unedited gel for Figure 5A

Tubulin

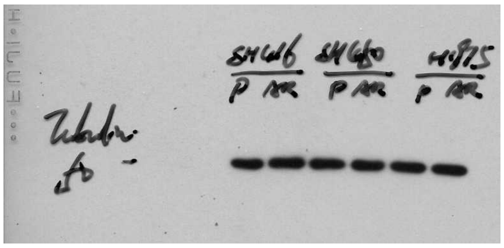

Full unedited gel for Figure 5B

TOP2α

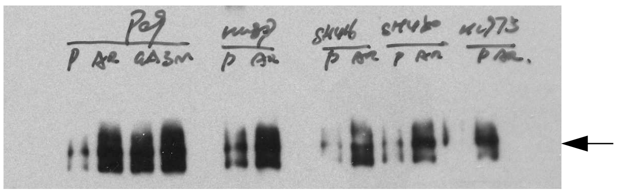

Full unedited gel for Figure 5F

FBXW7

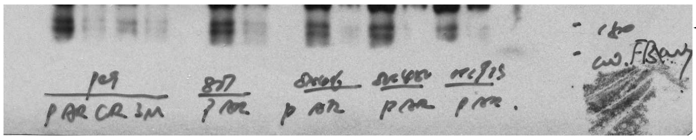

Full unedited gel for Figure 5F

Smurf2

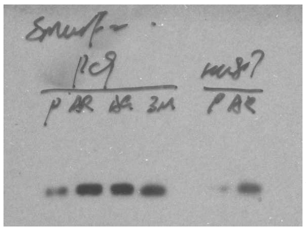

Full unedited gel for Figure 5F

Smurf2

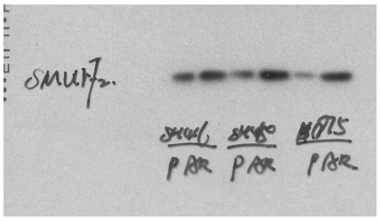

Full unedited gel for Figure 5F

Tubulin

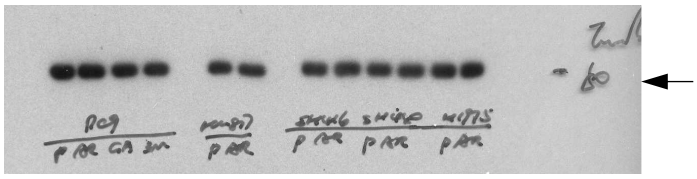

Full unedited gel for Figure 5F

TOP2α

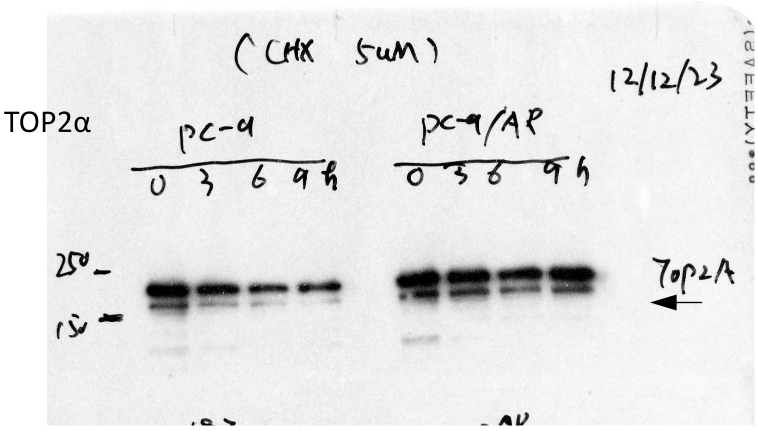

Full unedited gel for Figure 5H

Tubulin

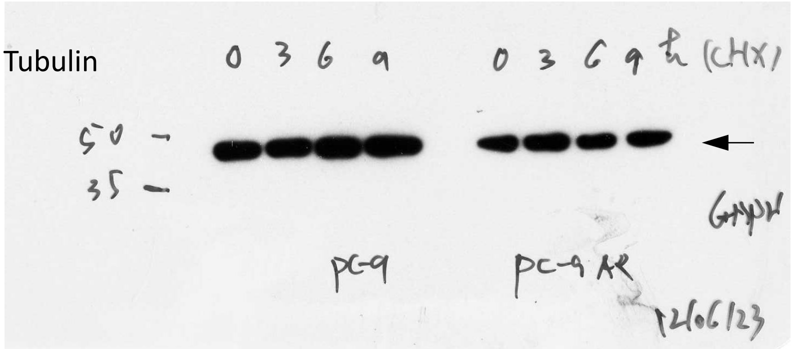

Full unedited gel for Figure 5H

Fig 5

PARP

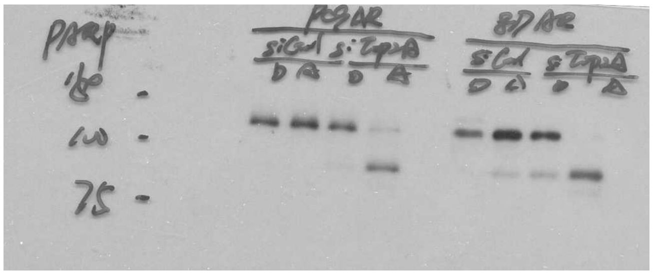

Full unedited gel for Figure 6A

TOP2α

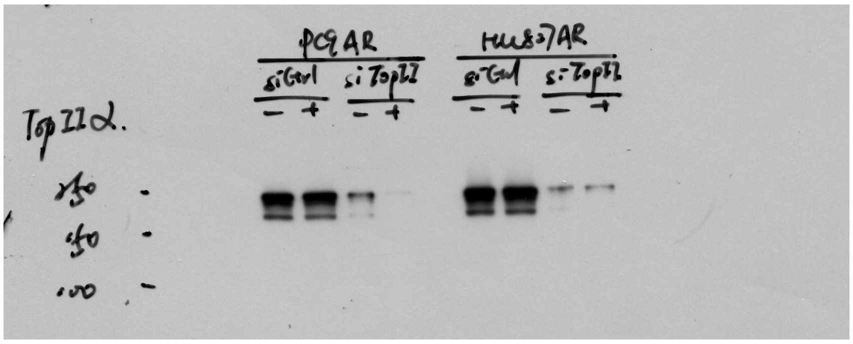

Full unedited gel for Figure 6A

Tubulin

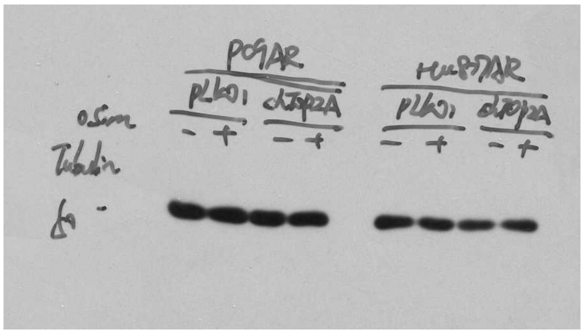

Full unedited gel for Figure 6A

PARP

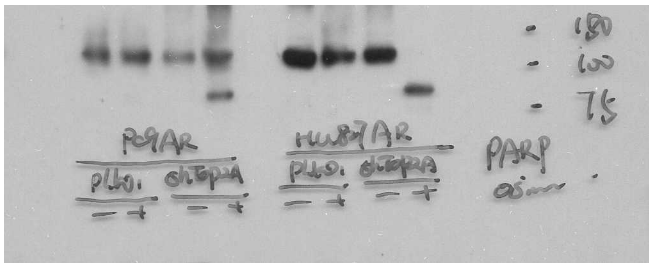

Full unedited gel for Figure 6B

TOP2α

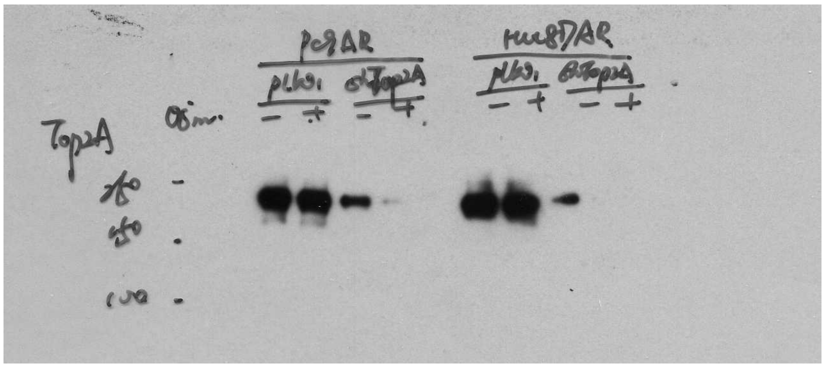

Full unedited gel for Figure 6B

Tubulin

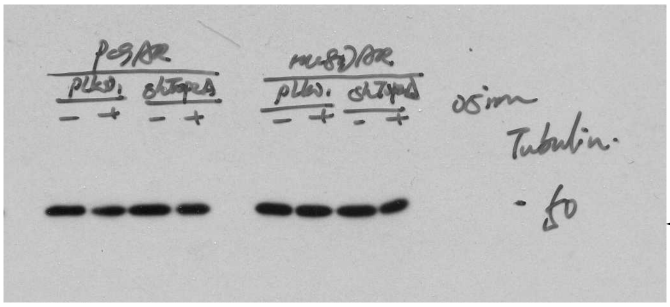

Full unedited gel for Figure 6B

Fig 6

TOP2α

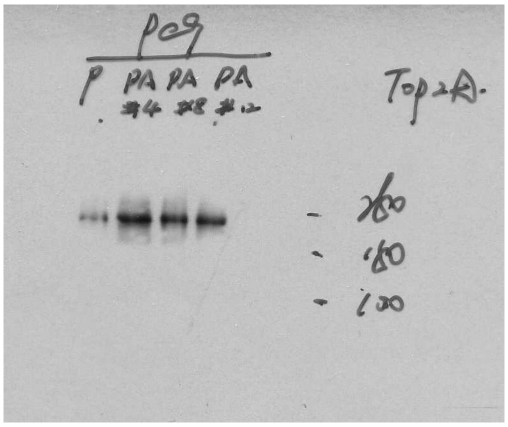

Full unedited gel for Figure 7A

Tubulin

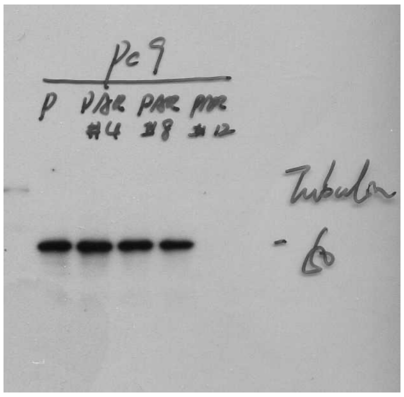

Full unedited gel for Figure 7A

PARP

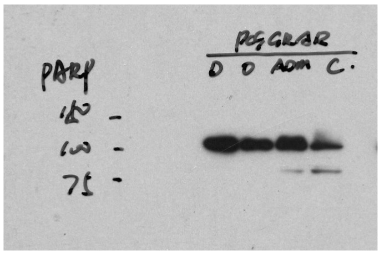

Full unedited gel for Figure S2D

Caspase 3

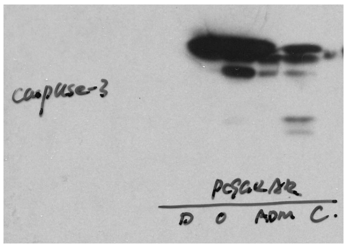

Full unedited gel for Figure S2D

Tubulin

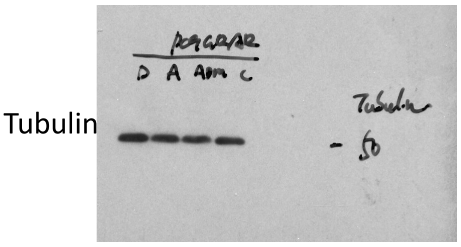

Full unedited gel for Figure S2D

Fig S2

PARP

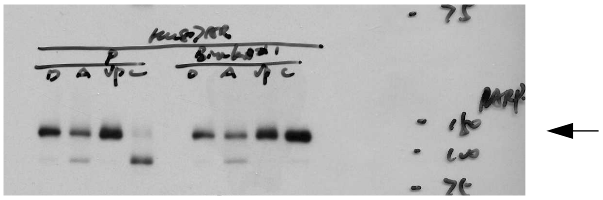

Full unedited gel for Figure S3A

Bim

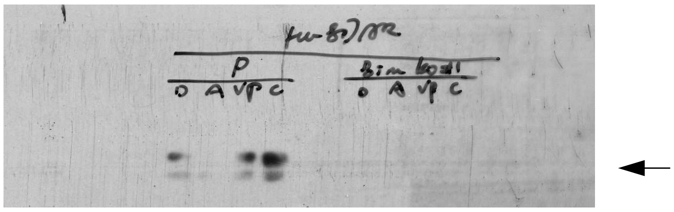

Full unedited gel for Figure S3A

Tubulin

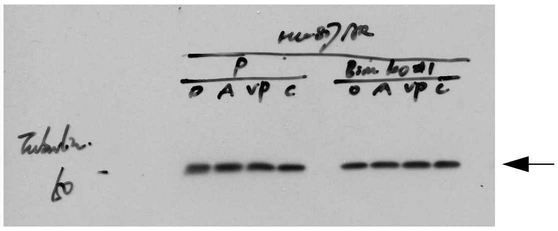

Full unedited gel for Figure S3A

TOP 2β

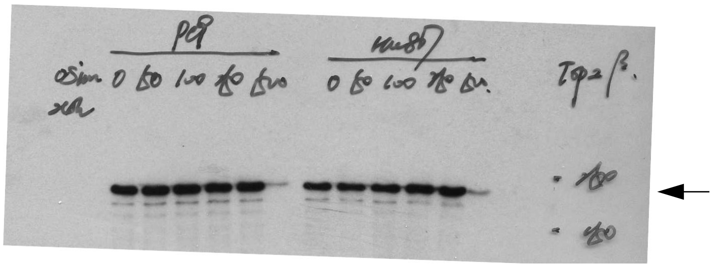

Full unedited gel for Figure S8

Tubulin

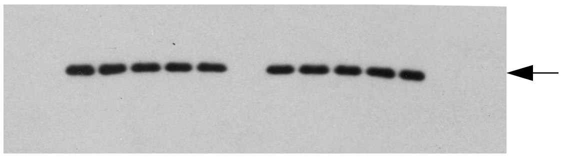

Full unedited gel for Figure S8

Fig S8

SMURF2

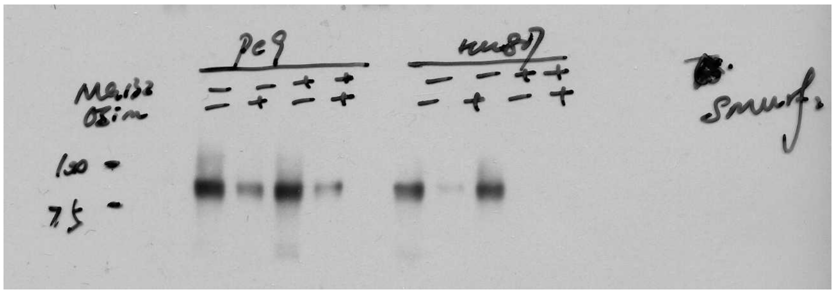

Full unedited gel for Figure S9

Tubulin

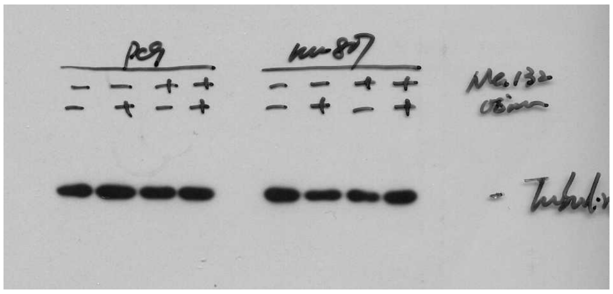

Full unedited gel for Figure S9
